# Thermostable Bacterial Esterases From Lipase Family 1.5 Degrade Compostable Polyesters PBAT and PBSA

**DOI:** 10.1002/mbo3.70144

**Published:** 2025-11-14

**Authors:** F. Hafna Ahmed, Lygie Esquirol, Nigel G. French, Raquel Aguiar Rocha, Pete Cass, Colin Scott

**Affiliations:** ^1^ CSIRO Environment, Black Mountain Laboratories Canberra Australian Capital Territory Australia; ^2^ Advanced Engineering Biology Future Science Platform, CSIRO Canberra Australian Capital Territory Australia; ^3^ CSIRO Manufacturing, Research Way Clayton Victoria Australia; ^4^ Enzide Technologies Ltd Perth Western Australia Australia; ^5^ University of Western Australia Crawley Western Australia Australia

**Keywords:** biodegradation, bioinformatics, environmental microbiology

## Abstract

The escalating plastics crisis, exacerbated by the accumulation of nonbiodegradable polyesters in the environment, has necessitated the exploration of sustainable waste management solutions such as enzymatic hydrolysis in industrial recycling. So far, the focus of these efforts has been on cutinase‐related polyethylene terephthalate (PET) degrading carboxylesterases, or PETases. In this work, we report the discovery and initial activity screen of previously uncharacterized, thermostable enzymes with polyesterase activity through comprehensive phylogenetic and sequence analysis of a bacterial family of esterases, Lipase Family 1.5. These enzymes are related to the previously identified polybutylene succinate co‐terephthalate (PBAT) degrading carboxylesterases Cl_EstA and Cl_EstB from *Clostridium botulinum* and PfL1 from *Pelosinus fermentans*. Originating from thermophilic bacteria, we show that these enzymes can be expressed heterologously in *Escherichia coli* and degrade the polyesters PBAT and polybutylene succinate co‐butylene adipate (PBSA), though they exhibit limited activity against PET. Notably, our results show that these enzymes are more effective at degrading the fully aliphatic polyester PBSA compared to the aliphatic‐aromatic co‐polyester PBAT, with three members of this enzyme family achieving complete solubilization of 5 mg/mL milled PBSA within 2 days at a low enzyme concentration (100 nM). This study highlights the substantial opportunity to find novel enzymes from nature that possess the required thermal stability for industrial applications, potentially reducing the need for extensive protein engineering.

## Graphical abstract

This study highlights that there is a broader diversity of enzymes with polyester depolymerase activity to be discovered from nature, beyond the well‐known PETases. We demonstrate that uncharacterized bacterial lipases possess polyesterase activity and thermostability, offering promising candidates for industrial plastic recycling without extensive protein engineering.

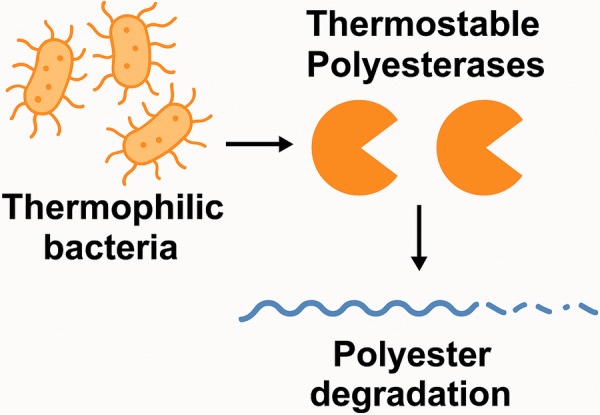

## 1 Introduction

From packaging to textiles, electronics, containers, and innumerable other applications, polyesters are integral to every aspect of modern life. The qualities that make polyesters attractive materials (durability and low reactivity) have also inadvertently created a global environmental disaster (Maity et al. 2021). Many polyester‐based products are intended for single use, but their complete degradation in landfill or water bodies can take centuries (Chamas et al. 2020). The low cost of polyester manufacture, compared to the cost of their recycling, along with improper waste disposal practices and the proliferation of small particulate ‘micro‐plastics’ has led to the steady accumulation of polyester waste in the environment (Worm et al. 2017; Lebreton et al. 2018; Rochman 2018; Moharir and Kumar 2019).

Unlike the inert carbon‐carbon backbones in polyolefin‐based plastics, the heteroatomic backbones in polyesters contain labile carboxyl ester bonds that, in principle, make them susceptible to hydrolysis and biodegradation (Shi et al. 2009). Aromatic polyesters such as polyethylene terephthalate (PET) are the most widely used due to their durability, but their hydrophobicity and crystallinity hinder hydrolysis and slows natural biodegradation (Mueller 2006). On the other hand, fully aliphatic polyesters, such as polybutylene succinate co‐butylene adipate (PBSA) and aliphatic‐aromatic co‐polyesters such as polybutylene succinate co‐terephthalate (PBAT) are considered “compostable” (Witt et al. 1999; Tserki et al. 2006), although their biodegradation rate is heavily affected by physiochemical conditions and microbial activity (Kijchavengkul et al. 2010; Puchalski et al. 2018).

Many fungi and bacteria that degrade polyesters enzymatically have been identified and often use the degradation products as carbon sources (Mueller 2006; Mohanan et al. 2020; Kaushal et al. 2021). In nature, complete polyester biodegradation is facilitated by their initial depolymerization using secreted hydrolytic enzymes such as cutinases, lipases, and esterases (Mohanan et al. 2020). These enzymes predate anthropogenic polyesters, having evolved to degrade natural polyester and carboxyl ester substrates, such as cutin (the waxy cuticle that protects the surface of plants) (Nikolaivits et al. 2018), esters of ω‐hydroxy fatty acids that can also contain aromatic groups like synthetic polyesters (Treichel et al. 2010), and a wide range of other naturally occurring soluble carboxyl esters (Bornscheuer 2002).

The biodegradability of polyesters presents the potential for chemical recycling of plastics (Feghali et al. 2020); in a circular economy, enzyme‐mediated depolymerization of polyester waste could recover the polyester building blocks as an input for new plastic synthesis (Wei and Zimmermann 2017; Tournier et al. 2023). However, this requires enzymes with higher catalytic efficiency than those found in nature (Wei and Zimmermann 2017). One method for increasing catalytic rates in enzyme‐mediated polyester depolymerization is to increase the incubation temperature above the polymers glass transition temperature (*T*_g_). At temperatures above *T*_g_ the crystallinity of some polyester is disrupted and for both crystalline and amorphous plastics this makes polymer chains more flexible and accessible for enzyme binding, thereby increasing chain mobility to facilitate enzymatic hydrolysis (Mueller 2006; Wei and Zimmermann 2017).

Enzyme engineering methods such as directed evolution (Bell et al. 2022), site‐directed mutagenesis (Furukawa et al. 2019; Tournier et al. 2020), and machine learning‐aided engineering (Lu et al. 2022) have been applied to improve polyester degradation activity of enzymes (Zhu et al. 2022), including their thermostability so that they can operate at temperatures that exceed the *T*_g_ of their target polyesters (Roth et al. 2014; Joo et al. 2018; Furukawa et al. 2019; Akram et al. 2024). Temperature‐stable enzymes are also inherently more resistant to protein unfolding and denaturation at ambient temperatures, which in turn improves protein expression yield, catalytic activity, as well as shelf‐life during storage and transport (Silva et al. 2018). In addition to protein engineering, polyester‐degrading enzymes with increased thermostability have been isolated from thermophilic micro‐organisms (Roth et al. 2014), as they tend to show greater inherent resistance to higher temperatures compared to enzymes isolated from mesophiles (Vieille and Zeikus 2001).

Two esterases from *Clostridium botulinum*, Cl_EstA and Cl_EstB, have been shown to hydrolyze the polyester PBAT (Perz et al. 2016). A third related protein, PfL1 from *Pelosinus fermentans*, with the same βα_8_ protein fold and 55% sequence identity to Cl_EstA was also reported to degrade PBAT (Biundo et al. 2016). All three proteins belong to bacterial lipase family 1.5 as classified in the ESTHER database of α/β hydrolase fold enzymes (Arpigny and Jaeger 1999; Lenfant et al. 2013; Chatonnet et al. 2023), along with closely related thermoalkalophilic and organic solvent tolerant lipases that have been characterized from *Geobacillus sp* (Schmidt‐Dannert et al. 1996; Kim et al. 1998; Nawani et al. 1998; Jeong et al. 2002). These enzymes possess a common catalytic triad composed of conserved serine, aspartic acid, and histidine residues, as well as two aspartic acid and two histidine residues comprising a conserved zinc coordination site (Jeong et al. 2002; Biundo et al. 2016; Perz et al. 2016; Timucin et al. 2016). In addition, the active site of these enzymes is protected from bulk solvent by an α‐helix that forms a “lid” structure and limits the accessibility of large substrates (Jeong et al. 2002; Biundo et al. 2016; Perz et al. 2016). The partial removal of this lid improves the catalytic rate of Cl_EstA with PET (Biundo et al. 2017).

Despite their similarity to Cl_EstA, Cl_EstB and PfL1, no other members of this family have been shown to have polyesterase activity (Schmidt‐Dannert et al. 1996; Kim et al. 1998; Nawani et al. 1998; Jeong et al. 2002). However, as this family of lipases includes highly stable enzymes, we decided to explore this family for thermophilic polysterases and perform an initial screen of their polyester degradation activity. We examined the phylogenetic distribution of enzymes of this family and characterized six uncharacterized homologs from thermophilic bacteria and one from an acidophile. We investigated their thermostability and substrate range and tested their ability to degrade PBAT, PBSA and PET. We found that multiple previously uncharacterized members of Lipase Family 1.5 exhibit significant thermostability and polyester‐degrading capabilities, particularly towards the compostable polyesters PBAT and PBSA.

## 2 Methods

### 2.1 Sequence Retrieval and Sequence Similarity Network (SSN) Generation

The sequences of Cl_EstA (Accession no: WP_011948553.1), Cl_EstB (Accession no: WP_011986581.1) and PfL1 (Accession no: EIW29778.1) were used to perform a pBLAST search against the NCBI Refseq_Select_proteins database (O'Leary et al. 2016) using default parameters. All sequences identified with e‐values > 0.005 were retrieved and submitted to the Enzyme Function Initiative Enzyme Similarity Tool (EFI‐EST) (Gerlt et al. 2015) to generate a sequence similarity network (SSN) that only contained sequences of lengths between 200 and 1000 amino acids, with an initial alignment score cut‐off of 7. In an SSN, nodes represent individual proteins and edges represent the alignment score, which is calculated by the EFI‐EST algorithm using the bit‐score from performing an all‐vs‐all BLAST of the provided protein sequences. The alignment score is close in magnitude to the negative logarithm of the BLAST *E‐value* that approximates protein similarity (Altschul et al. 1990; Gerlt et al. 2015).

**Figure 1:**
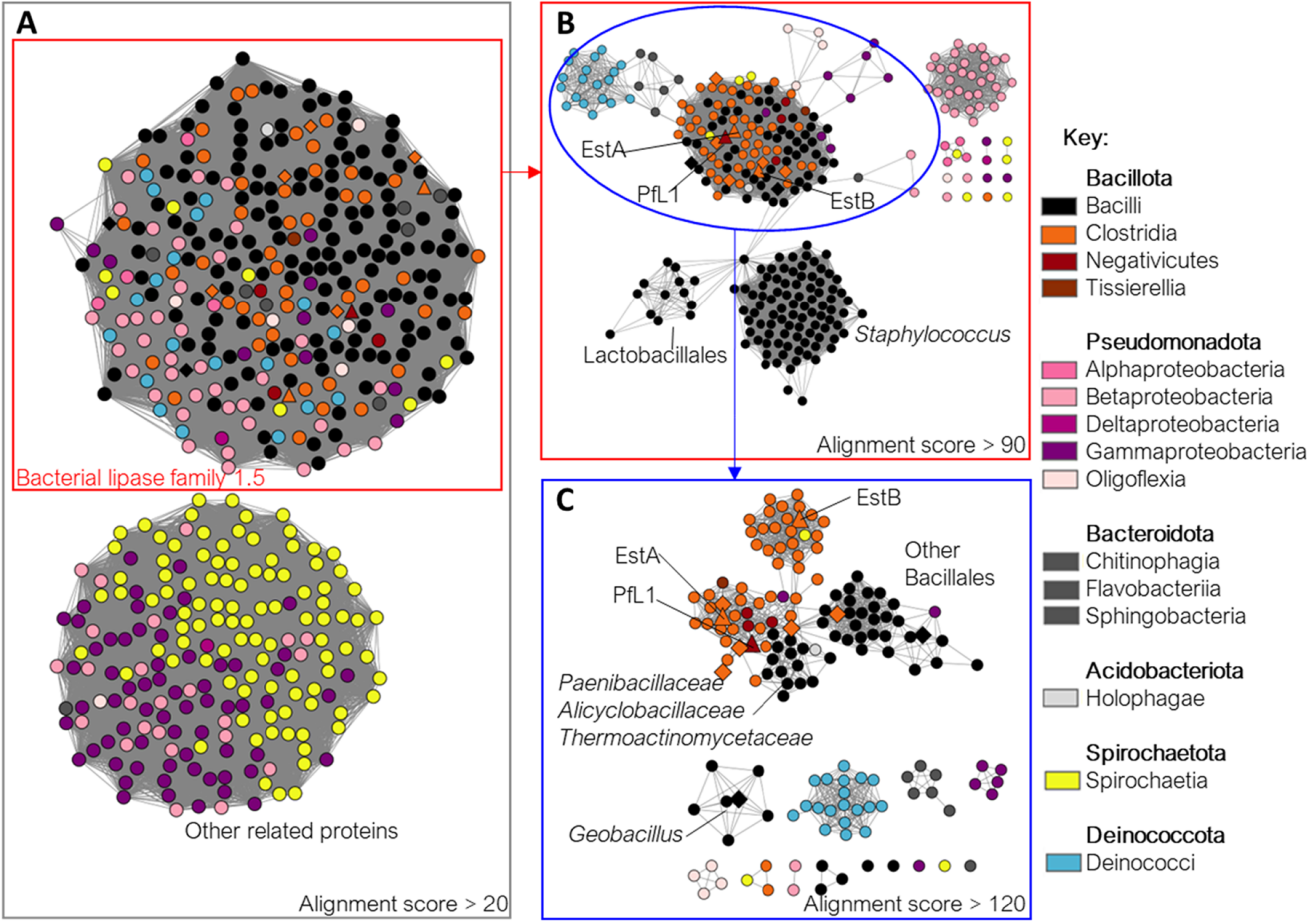
Sequence similarity network (SSN) of the bacterial lipase family 1.5 (Arpigny and Jaeger 1999; Lenfant et al. 2013; Chatonnet et al. 2023) that include Cl_EstA, Cl_EstB and PfL1. Each node represents an individual protein and edges between them represent the Alignment score calculated by EFI‐EST (Gerlt et al. 2015), which indicates protein similarity. Nodes are colored based on the taxonomy class as in the provided key. Cl_EstA, Cl_EstB and PfL1 are shown as triangles and the 7 other proteins characterized in this work are shown as diamonds. (A) The complete sequence set retrieved from pBLAST (Altschul et al. 1990) at an alignment score cut‐off of > 20 to isolate the cluster of proteins of the bacterial lipase family 1.5 from other related proteins. (B) The lipases at an alignment score cut‐off of > 90 showing the relatedness of proteins within this family based on their taxonomy. (C) The sequences in the encircled region of panel (B) at an alignment score cut‐off of > 120 showing the relationship between smaller clusters that include Cl_EstA, Cl_EstB and PfL1. In (B) and (C), Cl_EstA, Cl_EstB and PfL1 are labeled, as well as the order, family or genus for the large separate clusters from the class Bacilli.

The SSN was visualized using Cytoscape 3.9.0 (Shannon et al. 2003) by applying the yfiles Organic Layout. The clustering was analyzed by gradually increasing the alignment score cut‐off followed by reapplication of the layout until no major changes were observed with a large jump in the cut‐off value, which was from alignment scores of 20 through to 40 in this SSN (Supporting Information S1: Figure 1). This visual cluster analysis method allows the identification of potentially iso‐functional groups from other related proteins (Atkinson et al. 2009; Ney et al. 2017). Only sequences belonging to the large cluster that contained the query proteins were then used for further SSN and phylogenetic analysis. Further alignment score cut‐off values and subsequent yfiles organic layout reapplication was performed to visualize clades within the larger protein family.

### 2.2 Phylogenetic Analysis

The refined sequence set from the SSN was then aligned using the structure‐based protein sequence alignment algorithm PROMALS3D (Pei et al. 2008) with the structures of Cl_EstA (PDB ID: 5AH1 (Perz et al. 2016)) and PfL1 (PDB ID: 5AH0 (Biundo et al. 2016)) provided as templates. Poorly aligned regions at the N‐ and C‐terminus as well as long insertions in otherwise well aligned sequences were deleted, and sequences with poor overall alignment or large deletions were removed entirely. This curated sequence set was re‐aligned using MUSCLE (Edgar 2004) using the EMBL‐EBI webserver (https://www.ebi.ac.uk/Tools/msa/muscle/) with default settings. The resulting alignment was further trimmed to remove poorly aligned regions at the N‐terminus as well as any large gaps or insertions, before being used to generate an initial approximate maximum‐likelihood (ML) tree using FastTree2.1 (Price et al. 2010) with the WAG + CAT evolutionary model. The multiple sequence alignment (MSA) was further curated to remove any long branches along with highly similar sequences with very short branch lengths, and the process was repeated until the SH‐like local support values (Price et al. 2010) were > 80% for most nodes. The resulting alignment was used to infer a maximum likelihood tree using the IQ‐TREE webservice (https://www.hiv.lanl.gov/content/sequence/IQTREE/iqtree.html) (Trifinopoulos et al. 2016; Minh et al. 2020). The calculated best evolutionary model used was as follows: LG model + FreeRate heterogeneity (#rate categories = 8) + ML optimized AA frequencies from the data. Ultrafast bootstrap (UF‐Boot) values (Minh et al. 2013) and SH‐aLRT branch test values (Guindon et al. 2010) were calculated for 1000 replicates. The tree was visualized and annotated using the Interactive Tree Of Life (iTOL) webtool (https://itol.embl.de/) (Letunic and Bork 2021).

Sequences for further characterization were selected from known extremophile species represented in the phylogenetic tree, limiting to sequences from Clostridia and Bacilli of the order *Bacillales* as they were closest in homology to Cl_EstA, Cl_EstB and PfL1, with highest chance of similar enzymatic activity.

### 2.3 Protein Expression

The 7 identified protein sequences of interest along with the sequences of Cl_EstA, Cl_EstB and PfL1 were input into the SignalP‐5.0 webserver (Almagro Armenteros et al. 2019), and the signal sequence predicted was trimmed before designing the expression vectors that were ordered from GenScript (Singapore) (Supporting Information S1: Table 1). Each truncated sequence was placed between the Nde1 and Xho1 restriction sites in the expression vector pET‐29b(+) such that the expressed protein contains a C‐terminal 6xHistidine tag. These vectors were transformed into NEB T7 express cells (New England Biolabs) with the manufacturer recommended protocol and plated on Luria Broth (LB) agar plates containing 50 µg/mL of kanamycin. The plates were incubated overnight at 37°C and stored at 4°C for a maximum of 2 weeks. A negative control plasmid (gfasPurple‐S125R‐F162R‐V44A‐L123T_pETcc2 [Ahmed et al. 2022]) was also transformed and plated on an LB agar plate containing 100 µg/mL of ampicillin.

For protein expression, single colonies from each strain were inoculated into 10 mL of auto‐induction media (Tartof and Hobbs 1987) (5 g yeast extract, 20 g tryptone, 85.5 mM NaCl, 22 mM KH_2_PO_4_, 42 mM Na_2_HPO_4_, 0.6% glycerol, 0.05% glucose and 0.2% lactose) containing 50 µg/mL of kanamycin (100 µg/mL of ampicillin for the control strain) in 50 mL tubes. Cultures were grown for 3–6 h at 37°C while shaking at 200 rpm, followed by overnight incubation at 30°C. The resulting cultures were either stored at 4°C for up to 2 weeks for whole cell assays or spin down at 4000x g for 10 min at 4°C, where the supernatant was discarded, and the resulting pellet was stored at ‐20°C until protein purification.

### 2.4 Testing for Protein Expression With SDS‐Page

To test protein expression, 500 µL from each culture was spun down at 4000x g at 4°C for 10 min in 1.5 mL microfuge tubes and the supernatant was discarded. The resulting cell pellet was resuspended in 100 µL of lysis solution (50 mM Tris pH 8, 1x BugBuster Protein Extraction Reagent (Millipore) and ~33 nL DNAse I) and left on ice for about 10 min. After lysis, 5 µL from each sample was mixed with 10 µL of 50 mM Tris H 8 and 5 µL of 4x NuPAGE LDS Sample Buffer (Invitrogen). The rest of the samples were spun down at 20,000x g for 10 min at 4°C, and 15 µL of the supernatant was mixed with 5 µL of 4x NuPAGE LDS Sample Buffer (Invitrogen). The samples were heated at 90°C for 3 min and loaded onto pre‐cast NuPAGE^TM^ 4%–12% Bis‐Tris gels (Invitrogen) and run for 30–40 min at 150 V in MES SDS running buffer (Invitrogen). Gels were stained with AcquaStain Protein Gel Stain (Bulldog) for 30 min and de‐stained in water.

### 2.5 Partial Protein Purification and Enrichment

For the enrichment and partial purification of the proteins from small scale cultures, the pellets from 10 mL cultures were resuspended each in 1 mL of lysis buffer containing 50 mM Tris, 300 mM NaCl at pH 8 and transferred to 2 mL microfuge tubes. Cells were lysed by sonication (Misonix Sonicator 3000, 5 s pulse, 1 s break, 30 s, three times). The lysates were then spun at 20,000x g for 20 min and 4°C and the supernatant was loaded onto NEBExpress Ni Spin Columns (New England biolabs) that were pre‐washed with 250 µL of the same lysis buffer. The columns were washed with 750 µL total of wash buffer (50 mM Tris, 300 mM NaCl, 5 mM imidazole, pH 8) and the samples were eluted with 2x 200 µL of elution buffer (50 mM Tris, 300 mM NaCl, 500 mM imidazole, pH 8). From each eluent, 15 µL was mixed with 5 µL of 4x NuPAGE LDS Sample Buffer (Invitrogen) for SDS_PAGE analysis as described above. Purified proteins were stored at 4°C for up to 3 weeks. Because the proteins were not fully purified, the protein concentrations used in subsequent experiments were based on the extinction coefficient of the enriched protein and the absorbance of the enrichment solution at 280 nm according to the Beer–Lambert law.

### 2.6 Esterase Activity Assay With pNP‐Substrates

**Figure 2:**
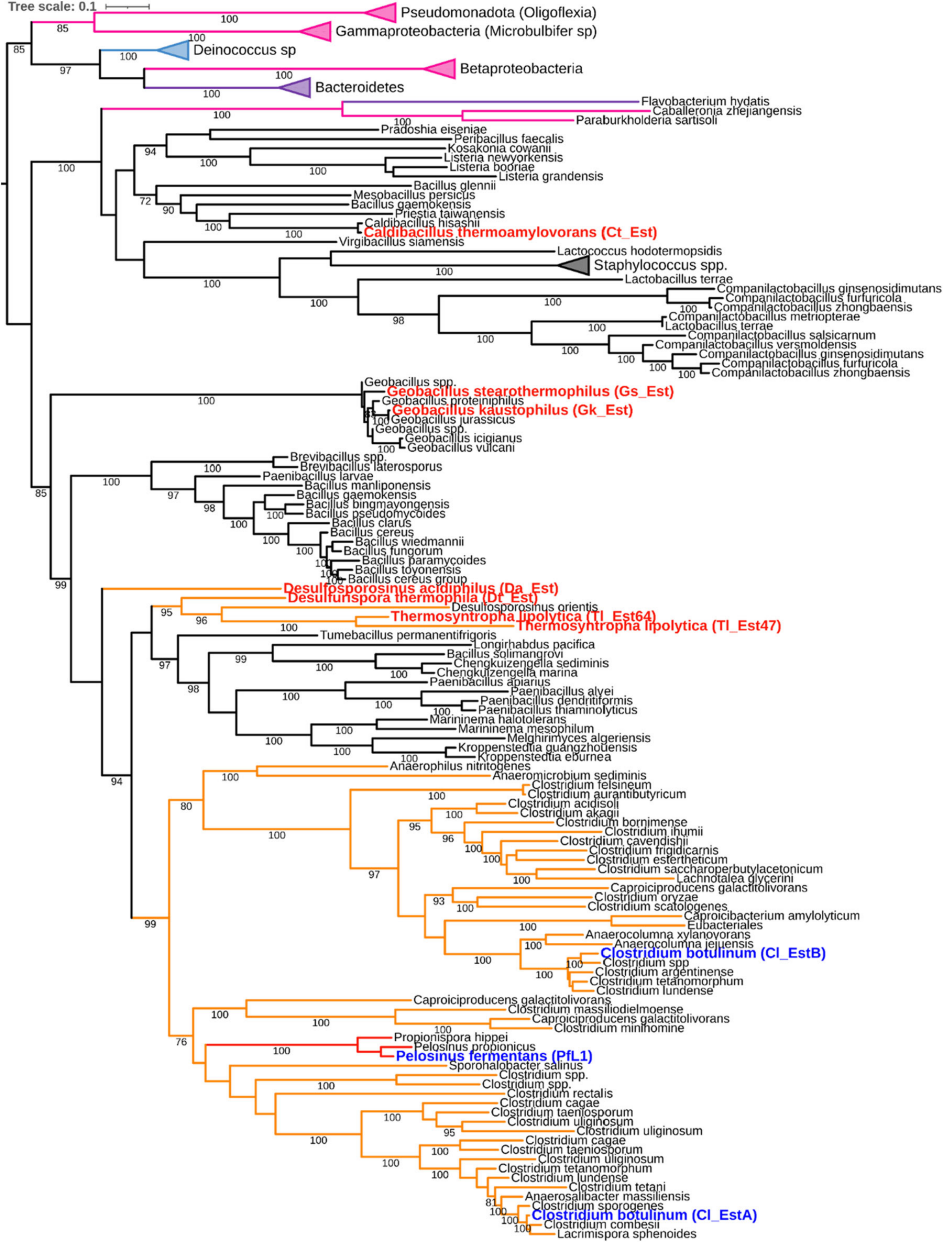
Unrooted maximum‐likelihood phylogenetic tree of the bacterial lipase family 1.5 generated using IQ‐tree (Minh et al. 2020) and visualized by iTOL v5 (Letunic and Bork 2021). UF‐Boot values are shown for nodes with SH‐aLRT support values >= 80%. Branches are colored as follows: *Clostridia* ‐ orange, *Bacilli* ‐ black, *Negetivicutes* ‐ red, *Psuedomonadota* – pink, *Bacteroidota* – purple, *Deinococcota* – light blue. Names of the previously characterized proteins Cl_EstA, Cl_EstB and PfL1 are labeled in the dark blue, and the new proteins characterized in this work are labeled in red. The tree containing sequence accession numbers and the expanded region containing sequences from *Psuedomonadota*, *Bacteroidota*, and *Deinococcota* are shown in Supporting Information S1: Figure 3.

To check the activity of the proteins with para‐nitrophenol (pNP) substrates, 90 µL of 50 mM Tris (pH 8.0) was first mixed with either 5 µL of cell culture for whole cell assays, or 5 µL of 200 nM purified protein. To start the reaction, 5 µL of 15 mM pNP‐acetate, pNP‐propionate, pNP‐butyrate, pNP‐velerate or pNP‐octanoate in 100% methanol was added, such that the final reaction contained 5% methanol. The absorbance at 405 nm was measured over 10 min at 3–4 s intervals and the rate at the linear portion of the curve (usually within the first 30–100 s) was used to calculate the amount of pNP produced as per a standard curve (Supporting Information S1:Figure 2).

### 2.7 Thermostability Assay

For each enzyme (and the negative control culture), 8x 15 µL of cell culture was added to 0.2 mL tubes in a strip and heated to various temperatures for 10 min using the temperature gradient function on a thermocycler (Kyratec Supercycler Thermal Cycler). The tubes were then cooled on ice for another 10 min. Residual activity was measured using the pNP‐substrate assay described above with either pNP‐acetate or pNP‐propionate. The specific temperatures used were 61.75°C, 63.25°C, 65.75°C, 69.25°C, 72°C, 75.25°C, 78°C, 81.75°C, and 84.25°C for pNP acetate assays and 65.75°C, 67.25°C, 69.75°C, 73.25°C, 76°C, 79.25°C, 82°C, 85.75°C, 87°C, 88.25°C, 89.75°C, 90.75°C, 93.25°C, 94.75°C, 95.09°C, 97.39°C, 98.77°C, and 99°C for pNP propionate assays. 5 µL of the heated and cooled cell culture samples were used, and a positive control containing 5 µL of unheated cell culture and a negative control containing 5 µL of buffer was also set up for each protein. Reaction rates in optical density per min were obtained and used to calculate the percentage residual activity as compared to the unheated positive control sample.

### 2.8 PBAT and PBSA Sample Preparation

Powdered samples of PBAT (SolPol 1000 N, supplied by Cosmos Plastics and Chemicals) and PBSA (BioPBS FD92, supplied by PTTMCC) were prepared by cryogenic milling (IKA Lab Mill) in liquid nitrogen. The samples were dried under vacuum at 50°C and sampled between 400 and 200 µm sieves.

### 2.9 PBAT and PET Degradation Assays

First, 10 mg/mL suspensions of PBAT or PET were prepared in a buffer containing 50 mM Tris pH 8.0. Then, 250 µL of the suspension was aliquoted into 1.5 mL or 2 mL tubes, one for each enzyme being assayed. For whole‐cell assays, an additional 250 µL of buffer was added, diluting the PBAT or the PET to 5 mg/mL, and 5 or 10 µL of cell suspension was added to start the reaction. For the assays with partially purified enzyme, 250 µL of approximately 200 nM solutions of each enzyme in the same buffer were added to the suspensions, such that the final concentrations of PBAT or PET was 5 mg/mL and enzyme was 100 nM. A negative control with just a buffer was also included. The reactions were incubated, shaking at either room temperature, 40°C, 70°C or 90°C for 24 or 48 h. For the assays with cell suspensions no additional cell lysis was performed as most of the cells are expected to have auto‐lysed either before the experiment due to nutrient depletion during growth to late stationary phase and prolonged storage at 4°C (Kabir et al. 2005; Cao‐Hoang et al. 2010) or during the experiment due to further nutrient depletion in the Tris buffer and incubation at 70°C (Packard et al. 2013).

At the end of the incubation period, the samples were spun down at 16,000x g for 1 min at room temperature to pellet the remaining plastics, and 2x 200 µL aliquots of the supernatant were transferred to a 96‐well UV‐plate (Grenier) from each tube. Absorbance at 250 nm was measured using a spectrophotometer (Molecular Devices Spectramax Plus 384), and the amount of terephthalic acid produced was measured as per a standard curve (Supporting Information S1: Figure 2). Values from technical replicates were averaged and 2 or 3 experimental repeats were performed to calculate the standard error from the mean (SEM). The background values from the negative control containing only buffer and substrate were subtracted from values for those containing enzyme.

### 2.10 PBSA Degradation Assays

The PBSA degradation assays were set up the same as described above. After aliquoting 2x 200 µL of from each tube into a 96‐well plate, 10 µL of 5 mM NAD^+^ and 5 µL of 4 U/mL Alcohol Dehydrogenase Equine (Sigma‐Aldrich) was added to an equal number of adjacent wells. Into these wells, 185 µL of each PBSA reaction aliquot was transferred and the production of NADH + H^+^ over time was measured at an absorbance of 340 nm using a spectrophotometer (Molecular Devices Spectramax Plus 384). The same process was also used to measure PBAT degradation activity in comparison with the UV absorbance measured as described above. Background values from negative controls containing only buffer and substrate were subtracted from the experimental values. A standard curve was generated using the same method with twofold diluted concentrations of 1,4‐butanediol, starting from 10 mM in the final reaction (Figure 6A). An allosteric sigmoidal curve was fitted to the data using Graphpad Prism and used to estimate the amount of 1,4‐butanediol produced during PBSA/PBAT degradation.

## 3 Results and Discussion

### 3.1 Relationships Between Cl_EstA, Cl_EstB, and PfL1 Related Proteins and Their Taxonomic Distribution

Initially, we retrieved sequences that are homologous with the polyesterases Cl_EstA, Cl_EstB and PfL1 to generate a sequence similarity network (SSN) and visualize their taxonomic distribution. SSNs allow the prediction of protein structure‐function relationships at relatively low computational cost, but without inferring evolutionary relationships, by clustering and connecting proteins sequences based on similarity (E‐value) (Atkinson et al. 2009; Uberto and Moomaw 2013).

A distinct cluster of proteins homologous to the Cl_EstA, Cl_EstB and PfL1 that belong to bacterial lipase family 1.5 (Arpigny and Jaeger 1999; Lenfant et al. 2013; Chatonnet et al. 2023) was identified with an alignment score cut‐off of 20 (Figure 1A). These sequences continued to cluster at greater stringency (alignment score cut‐off value of 40; Supporting Information S1: Figure 1). They are exclusively from bacteria, predominantly those that belong to the classes Bacilli and Clostridia (such as *C. botulinum* that produce Cl_EstA and Cl_EstB) from the phylum Bacillota (Figure 1). The next most abundant taxa belong to the classes Betaproteobacteria (phylum: Pseudomonadota) and Deinococci (phylum: Deinococcota).

By applying higher alignment score cut‐offs, the sequence similarity between proteins was further explored. At an alignment score cut‐off of > 90, EstA, EstB and PfL1 remain within a large cluster of proteins with a high sequence similarity, mostly from organisms of the classes Clostridia and Bacilli, including *Geobacillus sp* (Figure 1B), but separating from orthologs in other taxonomic groupings. This suggests that gene duplication and horizontal gene transfer events within this family of enzymes have been rare (but not absent).

At an alignment score cut‐off > 120, further relationships between the closest homologs of Cl_EstA, Cl_EstB, and PfL1 are revealed (Figure 1C). At this cut‐off, Cl_EstA and PfL1 appear more closely related to each other and belong to the same cluster, while Cl_EstB and its closest homologs form a second related cluster with representatives of organisms mostly from the class Clostridia. A third related cluster is also present with proteins from the class Bacilli. The Cl_EstA and PfL1 cluster almost exclusively contains sequences from Clostridia and Bacillus (Figure 1C), excluding the genus *Geobacillus* and the order Lactobacillales (Figure 1B), that form separate smaller clusters. The proteins from *Pelosinus sp* such as PfL1 from *P. fermentans* are the only representatives from the class Negetivicutes. Spore‐forming Negetivicutes such as *Pelosinus sp* are closely phylogenetically related to *Clostridia* with the same genomic distribution of sporulating genes and are particularly closely related to *C. botulinum* that produces Cl_EstA and Cl_EstB (Vesth et al. 2013; Yutin and Galperin 2013). This suggests that there has been at least one gene duplication event within this enzyme family during the evolution of the Bacillota since the separation of the Clostridia/Negetivicutes branch from the rest of the Bacilli. Overall, this suggests that gene duplication within the Lipase 1.5 family may be relatively recent.

The bacteria within the orders and classes noted in this analysis are predominantly soil‐dwelling, with many Bacillota such as *Clostridium, Geobacillus, Bacillus* and *Caldibacillus* isolated from compost (Aguilar‐Paredes et al. 2023). In addition, several species of *Clostridium, Bacillus, Deinococcus*, and *Geobacillus* are known to be thermophiles, and their encoding proteins have been adapted for various industrial applications (McMullan et al. 2004; Kumar et al. 2019; Basu 2022).

### 3.2 Selection of Potentially Thermophilic Enzymes From Lipase Family 1.5

We used the sequences curated using the SSN (Figure 1A) to generate a maximum‐likelihood phylogenetic tree (Figure 2) for Bacterial Lipase Family 1.5. This analysis supports the observations made from the SSN, where Cl_EstA, Cl_EstB and PfL1 belonged to a large clade containing proteins from bacteria of the *Clostridiaceae* family. Within this clade, two sub‐clades contain each Cl_EstA and Cl_EstB, suggesting that they could have arisen from a gene duplication event in the last common ancestor of *Clostridiaceae*. Consistent with the SSN, PfL1 appears more closely related to Cl_EstA than Cl_EstB. The sequences from *Clostridiaceae* are most closely related to those from Bacilli of the families *Thermoactinomycetaceae, Paenibacillaceae*, and *Alicyclobacillaceae* as also seen on the SSN (Figure 1C). Among these Clostridia and Bacilli are also sequences from thermophilic and acidophilic Clostridia of the families *Peptococcaceae* and *Syntrophomonadaceae*.

Separate phylogenetic clades were observed for *Bacillus sp* and *Geobacillus sp*, as well as a third more distant clade containing Bacilli of the orders *Bacillales* (such as *Bacillus sp* and *Caldibacillus sp*) and *Lactobacillales*. Almost all the sequences from other phyla except for Bacillota form a separate clade on the tree, except for three sequences from Betaproteobacteria and Bacteroidota that are likely to have been acquired through horizontal gene transfer.

Based on the phylogenetic tree and SSN, we limited further functional analysis to sequences from Clostridia and Bacilli of the order *Bacillales*, as they demonstrated the closest homology to Cl_EstA, Cl_EstB and PfL1. We selected sequences from 6 thermophiles and 1 acidophile namely: Ct_Est from *Caldibacillus thermoamylovorans* (accession: WP_152032401.1), Gk_Est from *Geobacillus kaustophilus* (accession: WP_044733155.1), Gs_Est from *Geobacillus stearothermophilus* (accession: WP_095860225.1), Tl_Est47 from *Thermosyntropha lipolytica* (accession: WP_073088947.1), Tl_Est64 from *Thermosyntropha lipolytica* (accession: WP_014826614.1), Dt_Est from *Desulfurispora thermophila* (accession: WP_018085325.1) and Da_Est from *Desulfosporosinus acidophilus* (accession: WP_014826614.1).

### 3.3 Heterologous Expression and Characterization of Selected Lipase Family 1.5 in *E. coli*

**Figure 3:**
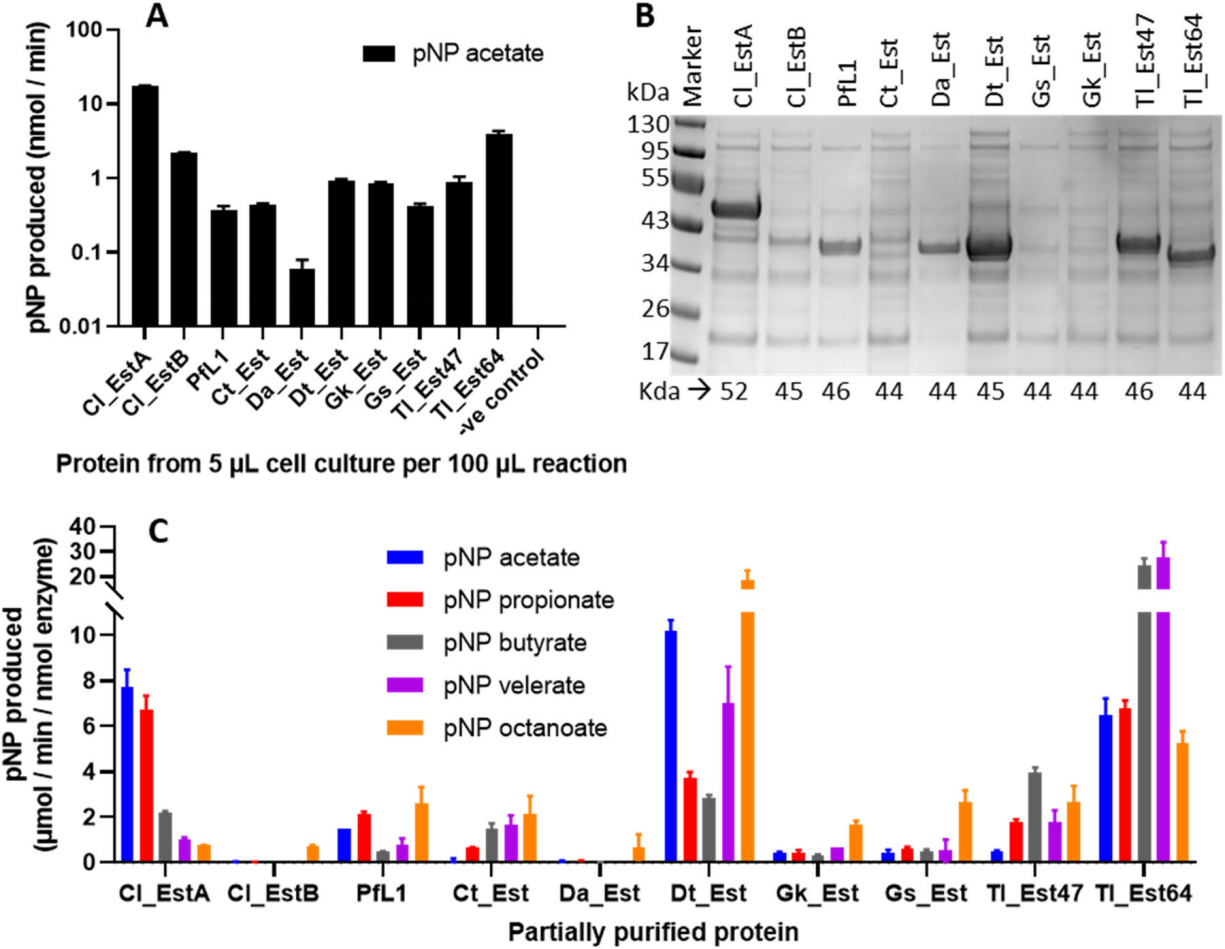
Esterase activity of the lipase homologs with pNP substrates. (A) Activity test using 5 µL of cell culture with 0.75 mM pNP acetate to confirm protein expression. Reactions were performed at room temperature and error bars represent SEM from three repeat experiments. (B) SDS‐PAGE gel analyzing the purity of the proteins obtained from small‐scale nickel affinity purification. The expected sizes of the bands are indicated at the bottom of the gel. (C) Comparison of the activity of approximately 10 nM of partially purified protein mixture with pNP substrates of different lengths. Error bars indicate SE from two repeat experiments performed at room temperature.

**Figure 4:**
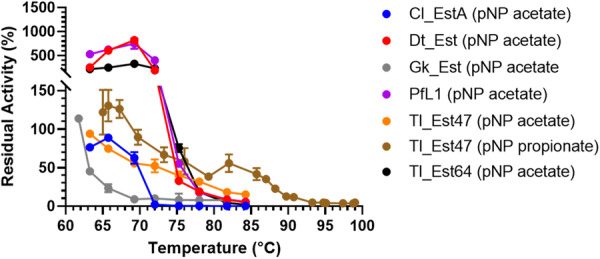
Residual activity of the thermostable proteins with pNP acetate and pNP propionate. Samples were heated at the designated temperature for 10 min and cooled at 4°C for at least 10 min before measuring the residual activity at room temperature, which is provided as a percentage of activity of an unheated sample. The 6 enzymes shown were identified from an initial assay with all 10 enzymes presented in this work (Supporting Information S1: Figure 5). Error bars represent SEM from three repeat experiments.

Selected Lipase Family 1.5 proteins were cloned for expression in *E. coli* strain NEB T7 Express. A C‐terminal His‐tag was added and signal peptides predicted by SignalP‐5.0 were removed (Almagro Armenteros et al. 2019), consistent with previous work on related lipases from *Geobacillus* sp. (Rúa et al. 1998; Kim et al. 2000). Protein profiles of the whole cell fraction (soluble and insoluble protein) and soluble fraction isolated from the cell cultures were evaluated on an SDS‐PAGE gel (Supporting Information S1: Figure 4). Clear bands were only observed for Cl_EstA, Cl_EstB, Dt_Est and Tl_Est64 due to a strong background band at the expected size that is also observed in the negative control. Esterase activity assays with p‐nitrophenyl (pNP) acetate (Figure 3A) were also conducted to confirm heterologous expression of active enzymes. Activity was detected for all 10 proteins but not the negative control, confirming their successful expression in *E. coli*.

The relative activities of the proteins were compared in an initial screen of enzyme activity. Proteins were partially purified by small‐scale nickel affinity chromatography (Figure 3B). Bands of approximately 90% purity or greater were observed for Cl_EstA, PfL1, Da_Est, Dt_Est, Tl_Est47 and Tl_Est64. Although only ~40–50% purity was achieved for Cl_EstB and < 10%–20% for Ct_Est, clear bands for the protein of the expected sizes were still observed. In contrast, the sequences from *Geobacillus sp*, Gs_Est and Gk_Est, had barely detectable protein purified using this protocol, even though pNP acetate activity was observed from the whole cell samples. This was unexpected since homologs from other *Geobacillus species* such as *G. stearothermophilus L1* (GenBank accession: AAC12257.1) (Kim et al. 2000; Jeong et al. 2002), and *G. thermocatenulatus* (previously *Bacillus thermocatenulatus*, GenBank accession: Q59260) (Schmidt‐Dannert et al. 1996; Rúa et al. 1998), generated relatively high expression in *E. coli* BL21DE3 cells. However, these studies did not include a C‐terminal His‐tag in their constructs like in this work, which may have affected protein expression levels.

The substrate ranges of the partially purified proteins were investigated, comparing activities with substrates with two to eight carbon chain lengths: pNP acetate, pNP propionate, pNP butyrate, pNP valerate and pNP octanoate (Figure 3C). Cl_EstB, Ct_Est, Gk_Est and Gk_Est were poorly enriched, while they showed very low activity with all the tested substrates. However, as previously observed for PfL1 (Biundo et al. 2016), a general trend of increased activity with increasing substrate length was observed for all proteins, except Cl_EstA, which had the best activity with pNP acetate consistent with the observations of Perz et al (Perz et al. 2016). Dt_Est showed the highest activity with pNP octanoate (albeit no activity with any other substrate; Figure 3B), which was the longest soluble substrate tested. Tl_Est64 showed the highest overall activity with the substrates pNP butyrate and pNP valerate.

### 3.4 Cl_EstA, PfL1, Dt_Est, Tl_Est47, and Tl_Est64 Are Highly Thermostable

**Figure 5:**
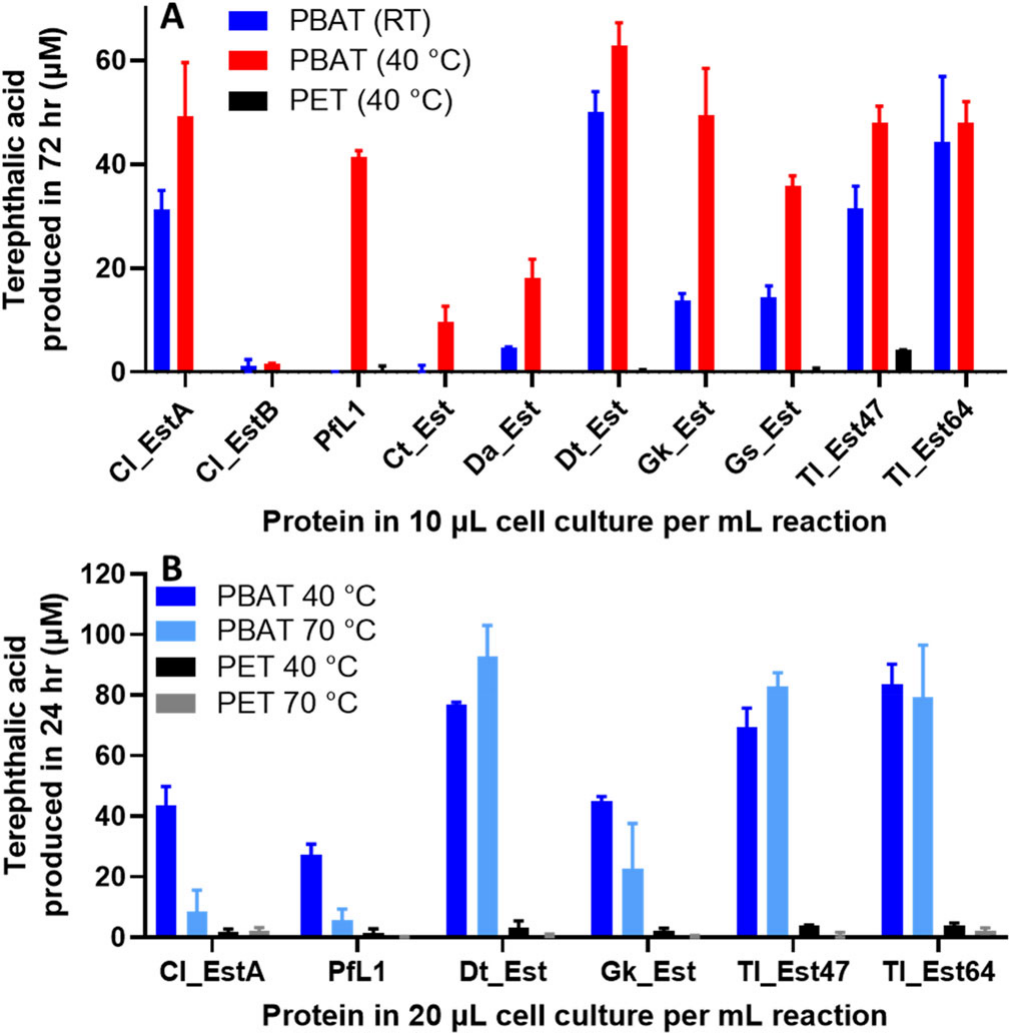
Polyesterase activity of the lipase homologs with PBAT and PET. (A) Activity of cell content expressing the lipase homologs over 72 h with PBAT at room temperature and 40°C, and with PET at 40°C. (B) Activity of cell content expressing the most thermostable lipase homologs over 24 h with PBAT and PET at 40°C and 70°C. Error bars indicate SEM from two repeat experiments and background levels of activity in the negative control containing only buffer and PBAT/PET have been subtracted. For both experiments, the cell suspensions were diluted into 50 mM tris (pH 8.0) from cultures grown to late stationary phase and stored at 4°C for 1–2 weeks and hence most cells are expected to have lysed before or during the experiment as detailed in Materials and Methods.

We tested the thermostability of the selected enzymes by testing the residual activity of cell cultures after heating at temperatures ranging from 55°C and 100°C for 10 min. An initial test demonstrated clear residual activity with pNP acetate for Cl_EstA, PfL1, Dt_Est, Gk_Est, Tl_Est47 and Tl_Est64 after heating at more than 60°C (Supporting Information S1: Figure 5). A more detailed evaluation showed that Dt_Est, PfL1, and Tl_Est64 retained over 10% residual activity even after heating at 78°C, Cl_EstA retained more than 50% residual activity after heating at 69°C, and Gk_Est showed more than 20% residual activity after heating at 66°C (Figure 4; Supporting Information S1: Table 2). Remarkably, Tl_Est47 retained over 10% activity with pNP acetate after heating at 84°C. When further tested with its preferred substrate, pNP propionate (Figure 3), Tl_Est47 displayed more than 10% residual activity after heating at 90°C, making it the most thermostable enzyme identified in this study. Interestingly, Dt_Est, Pfl1, and Tl_Est64 showed 500–1000% relative activity compared to the room temperature control reaction after incubation at 60°C–72°C, the exact reason for which remains to be further investigated. One reason for this could be that the higher temperatures promote the folding and activates previously inactive unfolded protein present in the cell suspensions used in these assays. The detailed mechanism of thermal stability including if these proteins prefer higher temperatures for improved protein folding and activity remains to be investigated.

These findings are consistent with previously characterized proteins within Lipase Family I.5. For example, Lipase L1 from *G. stearothermophilus L1* and BTL2 from *G. thermocatenulatus* have highest activity between 60°C and 70°C and melting temperatures (*T*_m_) of 69°C and 74°C, respectively, in the presence of zinc (Schmidt‐Dannert et al. 1996; Kim et al. 2000; Choi et al. 2005; Timucin et al. 2016). In these studies, their T_m_ values dropped significantly to 51°C and 65°C, respectively, without zinc supplementation. Our experiments (conducted without supplemental zinc) showed comparable thermostability of closely related enzymes from *Geobacillus*, where Gk_Est from *G. kaustophilus* (91% sequence identity to Lipase L1) lost activity after heating beyond 70°C, and Gs_Est from *G. stearothermophilus* (95% sequence identity to Lipase L1) showed no residual activity after heating at 56.5°C. Apart from increased zinc concentrations, increased ion‐pair interactions and hydrophobic residues in the protein lid region are thought to increase thermostability in this family of lipases (Ruslan et al. 2012; Timucin and Sezerman 2013). These may be factors that contribute toward the differences in thermostability observed between these tested proteins.

### 3.5 Polyesterase Activity in Lipase Family 1.5

**Figure 6:**
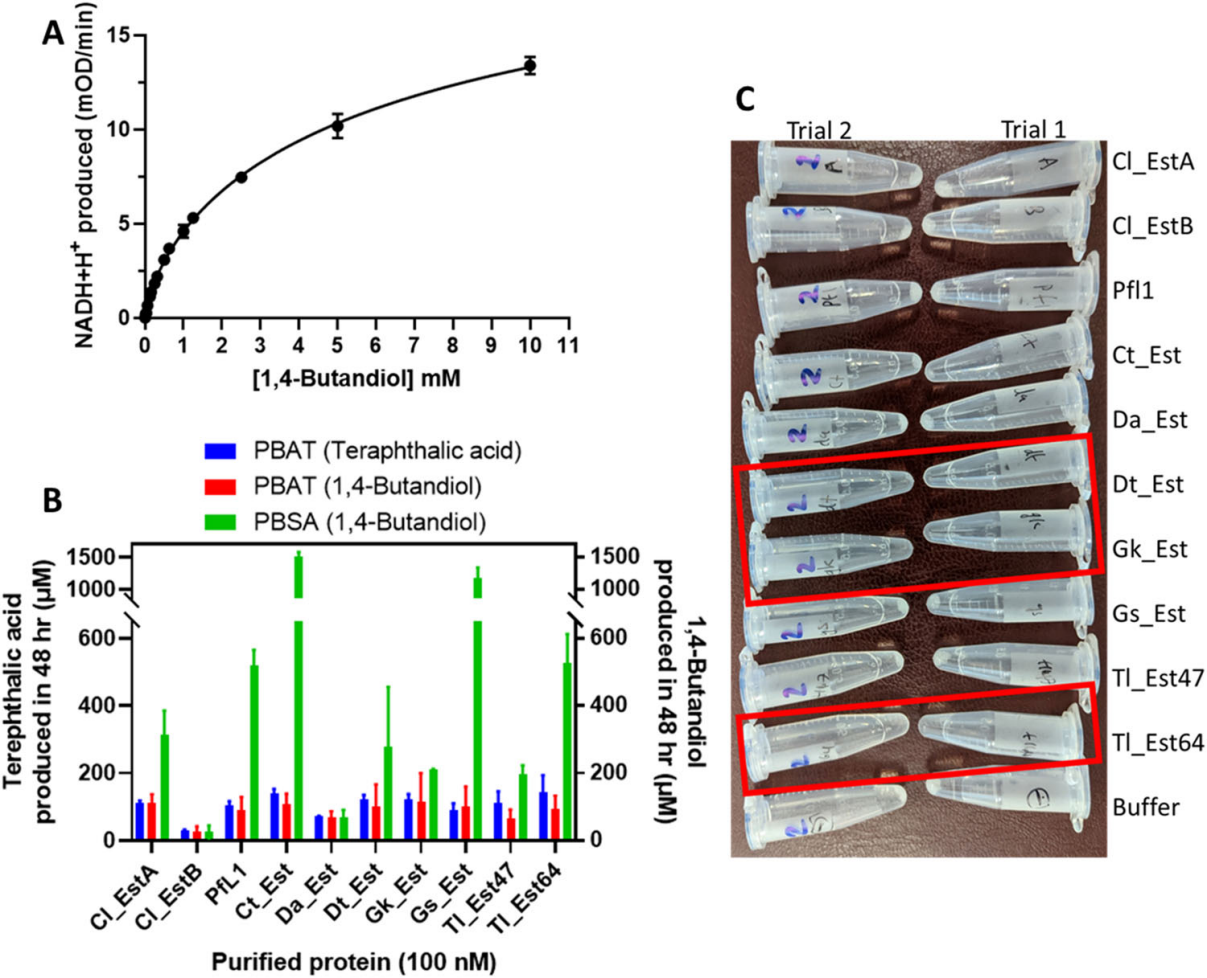
Polyesterase activity of the lipase homologs with PBAT and PBSA. (A) Standard curve for EqAD activity with 1,4‐butanediol. (B) PBAT and PBSA activity with 100 nM of partially purified protein at 40°C, measuring either the release of soluble terephthalic acid (and derivatives) or 1,4‐butanediol (and derivatives). Error bars indicate SEM from two repeat experiments. (C) Pellets of PBSA left after the reactions in (B), showing the complete degradation of the 5 mg/mL PBSA pellet by Dt_Est, Gk_Est and Tl_Est64, which are highlighted by red boxes.

We next investigated polyester degradation activity of enzymes from Lipase Family 1.5 using fine‐milled (< 0.5 mm) commercially available substrates, specifically amorphous PET, PBAT, and PBSA. PET degradation activity can be detected using “bulk” UV‐spectroscopy to measure the UV absorbance (200–300 nm) of the aromatic groups present on its depolymerization products; terephthalic acid, mono(2‐hydroxyethyl) terephthalate, and bis(2‐hydroxyethyl) terephthalate (Zhong‐Johnson et al. 2021) (Supporting Information S1: Figure 6). We reasoned that a similar method can be used to detect the products of enzymatic PBAT degradation, which have previously been shown to produce monomers of aromatic terephthalic acid, 1,4‐butanediol, and adipic acid, as well as longer oligomers of different sizes consisting of combinations of these three monomers (Jia et al. 2021). Initially, we incubated 5 mg/mL suspensions of PBAT with cell culture expressing Cl_EstA that has previously been confirmed as a PBAT hydrolase using reversed‐phase high‐performance liquid chromatography (RP‐HPLC) (Perz et al. 2016). We observed a clear time‐dependent increase in UV absorbance at approximately 250 nm over 1 week, confirming PBAT hydrolase activity detection via this method (Supporting Information S1: Figure 7).

Subsequent assays for PET and PBAT degradation with the full enzyme set were conducted for 72 h at room temperature and 40°C (Figure 5). A temperature of 40°C was chosen as it is close to the 37°C incubation temperature in previously reported experiments for Cl_EstA and Cl_EstB with PBAT and PBAT derived model substrates (Perz et al. 2016). At 40°C, PBAT degradation activity was observed with all enzymes, an at room temperature, all except PfL1 and Ct_EstB demonstrated activity. Notably, Cl_EstB showed low activity at both temperatures, consistent with prior RP‐HPLC studies (Perz et al. 2016). Conversely, amorphous PET degradation was negligible at 40°C, detected only weakly for Tl_Est47. This lack of activity was not unexpected since this incubation temperature is below the *T*_g_ of PET. Further testing at the elevated temperature of 70°C with the thermostable enzymes (Cl_EstA, PfL1, Dt_Est, Gk_Est, Tl_Est47, and Tl_Est64) revealed no significant PET degradation. Incubation of PETases with PET at temperatures exceeding the glass transition of PET ( ~ 65°C) has been shown to significantly enhance the PET hydrolysis rate (Akram et al. 2024); this was not observed for the Lipase Family 1.5 enzymes tested here, suggesting these enzymes either lack PET hydrolase activity or exhibit activity levels too low for detection.

Consistent with our findings, previous work showed that Cl_EstA only released very small amounts of PET hydrolysis products and that Cl_EstB did not show any detectable PET hydrolysis activity, even when higher activity levels were observed with PBAT and PBAT derived polymers (Perz et al. 2016). PfL1 also did not show any detectable PET hydrolysis activity (Biundo et al. 2016), and Cl_EstA did not show activity with the PET derived oligomer mono‐(2‐hydroxyethyl) terephthalate (MHET) (Biundo et al. 2017). Improvement in PET and MHET hydrolysis was observed for the Cl_EstA variant Δ71 with the N‐terminal “lid” region removed that otherwise covers the active site and restricts substrate binding (Biundo et al. 2017). The removal of the N‐terminal region also increased the overall surface hydrophobicity of Cl_EstA‐Δ71 compared to the wild‐type enzyme, which was hypothesized to increase the surface absorption of the enzyme to PET, leading to increased activity (Biundo et al. 2017). Hence, the underlying reason for the increased efficiency of these enzymes in hydrolyzing PBAT compared to PET may reflect the increased crystallinity and low accessibility of the PET polymers for hydrolysis due to its increased aromaticity, compared to the less crystalline PBAT aromatic‐aliphatic co‐polymer. Further work in computational modelling can delve into the molecular mechanisms behind the substrate specificity of these enzymes towards different polyester polymers.

To further assess the polyesterase specificity of these enzymes, we tested enzyme activity against PBSA, the degradation products of which – 1,4‐butanediol, succinic acid, adipic acid oligomers (Ando et al. 1998; Lee et al. 2008) – lack aromatic UV‐absorbing groups (Supporting Information S1: Figure 6). Hence UV spectroscopy could not be used to detect enzymatic degradation of PBSA, necessitating the development of an alternative assay.

We established an assay employing equine alcohol dehydrogenase (EqAD) to measure total alcohol production from PBSA hydrolysis via NAD^+^ reduction to NADH + H^+^ (absorbance at 340 nm; Figure 6A). EqAD is a homodimeric enzyme with a broad substrate range (Pietruszko et al. 1973; Matos et al. 1985), and our initial tests showed activity with different concentrations of 1,4‐butanediol, producing the expected allosteric sigmoidal kinetic curve for a homo‐dimeric enzyme. We then set up reactions containing 5 mg/mL PBAT with all the enzymes and compared the readout from measuring EqAD activity and measuring the UV absorbance, to detect both the 1,4‐butanediol and terephthalic derived products, respectively (Figure 6B; Supporting Information S1: Figure 8). We found that while the UV absorbance method was more reproducible between experiments with tighter error bars, the EqAD method produced comparable results, albeit with a wider spread of data points from experimental repeats.

We then set up equivalent reactions with PBAT and PBSA and measured the EqAD activity of 100 nM enzyme (Figure 6B). We found that all the enzymes could degrade PBSA, and except for Cl_EstB and Da_Est, the PBSA degradation activity was better than that observed for PBAT when assessed by EqAD activity. This is consistent with the lower crystallinity of PBSA polymers compared to PBAT due to being a fully aliphatic polymer compared to PBAT that is an aromatic‐aliphatic co‐polymer, which directly affects their enzymatic biodegradation (Tserki et al. 2006).

Interestingly, the reactions containing 100 nM of Dt_Est, Gk_Est, and Tl_Est64 showed a clear decrease in the leftover PBSA pellet with complete solubilization of the solid substrate after 48 h (Figure 6C). The same visually observable pellet loss was not seen in the PBAT degradation assay for these enzymes. However, the PBSA degradation reactions with these enzymes did not exhibit the highest level of activity using the EqAD assay. One explanation for this could be that there may be product inhibition of EqAD due to high rates of PBSA degradation and product accumulation. This means that this assay for PBSA degradation should be used in conjunction with observation of the reactions and the read‐out is only a qualitative estimate for the presence of PBSA degradation rather than being fully quantitative.

Another reason for this discrepancy is that these enzymes may not produce the monomeric 1,4‐butanediol product of the reaction that is detected using this analysis method. Rather, water‐soluble oligomers could be produced, even by the enzymes that solubilize PBSA completely. For example, an examination of the soluble reaction products of enzymatic PBSA degradation using NMR and GPC showed that commercially available lipases and cutinases with polyesterase activity produce a mixture of soluble oligomers along with the monomeric 1,4‐butanediol product (Rosato et al. 2022). While the coupled assay for PBAT and PBSA degradation activity that we report here, along with qualitative observation of plastic solubilization, gives an indication of the polyesterase activity of enzymes as an initial screen, further work is required for the detailed characterization of the reaction end‐products and quantification of the activities of these enzymes.

In these assays, Dt_Est, Tl_Est47, and Tl_Est64 showed thermotolerance, with similar levels of PBAT degradation at both 40°C and 70°C. This is consistent with the increased thermal stability of these proteins, where they retain more than 100% residual activity after heating at 70°C for 10 min (Figure 4). Although PfL1 also showed no loss of residual activity after heating at 70°C (Figure 4), it showed loss of PBAT degradation activity at 70°C compared to 40°C (Figure 5). This suggests a lower thermal tolerance for PfL1 compared with Dt_Est, Tl_Est47, and Tl_Est64, which was not fully detected in the residual activity assay, which can be investigated in further work by comparing the thermal stability over time of these enzymes at higher temperatures. Cl_EstA and Gk_Est also showed lower PBAT degradation at 70°C compared to at 40°C, consistent with loss of residual activity for both these enzymes after heating at 70°C for 10 min (Figure 4). Notably, the more thermostable enzymes also demonstrated higher overall PBAT degradation activity by ~2‐fold at 40°C. This could be because the improved enzyme stability leads to more sustained activity over the 24 h assay period. A similar effect was seen with an engineered variant of the polyester degrading enzymes jmPE13 and jmPE14 from *Pseudomonas* sp. JM16B3, where jmPE13 with a longer half‐life life at 40°C showed better PET and PBAT hydrolysis activity over 24 h (Zhou et al. 2024). A mutant of jmPE13 with improved enzyme half‐life at 40°C also showed increased PET and PBAT hydrolysis at both 40°C and 30°C over 90 h.

## 4 Conclusion

In this work, we have identified novel, naturally occurring thermophilic polyesterases that degrade the compostable polyesters PBAT and PBSA, but not PET. These enzymes are more effective in hydrolyzing the aliphatic polyester PBSA than the aliphatic‐aromatic co‐polyester PBAT. Three of the enzymes identified (Dt_Est, Gk_Est, and Tl_Est64) can completely solubilize a 5 mg/mL suspension of PBSA in less than 48 h at a concentration of 100 nM enzyme. Further investigations can elucidate the exact mechanism of thermotolerance of these enzymes, their detailed reaction mechanisms including their polyester degradation products and optimal reaction conditions.

The family of bacterial enzymes studied here are unrelated to the cutinase‐related polyesterases (Taniguchi et al. 2019), which are the subject of intense investigation due to their PETase activities. This suggests that polyester‐degrading activities may be found in a broad range of carboxylesterase families, and that there is still significant potential to identify new, naturally occurring, thermally tolerant enzymes from publicly available genomic data. Such enzymes can potentially be used in industrial applications without needing significant protein engineering for increased stability. While experimental confirmation of such activities will be necessary, the cost of database screening to identify candidate polyesterases can be reduced by Artificial Intelligence and Machine Learning approaches that define substrate scope and melting temperatures of uncharacterized enzymes.

## Author Contributions

**F. Hafna Ahmed:** conceptualization, methodology, data curation, investigation, validation, formal analysis, visualization, writing – original draft, writing – review and editing. **Lygie Esquirol:** data curation, investigation, validation, formal analysis, writing – review and editing, methodology. **Nigel G French:** data curation, investigation, methodology. **Raquel Aguiar Rocha:** methodology, writing – review and editing. **Pete Cass:** conceptualization, funding acquisition, project administration, resources, writing – review and editing. **Colin Scott:** conceptualization, methodology, data curation, investigation, formal analysis, supervision, funding acquisition, writing – original draft, writing – review and editing, resources, project administration.

## Ethics Statement

The authors have nothing to report.

## Conflicts of Interest

Pete Cass is employed by Enzide Technologies Ltd that funded this work.

## Supporting information


**SI Figure 1.** Initial prediction of iso‐functional homologues of Cl_EstA, Cl_EstB, and PfL1 using a sequence similarity network (SSN). **SI Figure 2.** Standard curves for p‐nitrophenol (pNP) and terephthalic acid used in this work. Reaction volumes were 100 μL for pNP and 200 μL for terephthalic acid. **SI Figure 3.** Phylogenetic tree showing sequence accession numbers for the enzymes analyzed. **SI Figure 4.** SDS‐PAGE gels analyzing protein expression in *Escherichia coli*. **SI Figure 5.** Initial tests assessing thermostability of all enzymes from cell cultures. **SI Figure 6.** End‐point hydrolysis products for the polyesters tested in this study. **SI Figure 7.** Measurement of bulk UV absorbance to detect PBAT degradation. **SI Figure 8.** EqAD activity measured in 185 μL of supernatant from a reaction containing 100 nM Cl_EstA and 5 mg/mL PBAT, incubated at 40°C for 48 hours. **SI Table 1.** Gene sequences for the proteins expressed in this study. **SI Table 2.** Statistical comparison of residual activity at key temperatures (69.25°C, 78.00°C, and 84.25°C) shown in Figure 4. Newly identified enzymes were compared to Cl_EstA using Welch's two‐tailed t‐tests (unequal variance, n = 3).

## Data Availability

The data that support the findings of this study are available from the corresponding author upon reasonable request.
