# Supplementary material for: Thermostable Bacterial Esterases From Lipase Family 1.5 Degrade Compostable Polyesters PBAT and PBSA
Source: Microbiologyopen. 2025 Nov 14;14(6):e70144. doi: 10.1002/mbo3.70144 (PMC12616515; doi:10.1002/mbo3.70144)
Supplement: Supplementary file 1 — SI Figure 1. Initial prediction of iso‐functional homologues of Cl_EstA, Cl_EstB, and PfL1 using a sequence similarity network (SSN). SI Figure 2. Standard curves for p‐nitrophenol (pNP) and terephthalic acid used in this work. Reaction volumes were 100 μL for pNP and 200 μL for terephthalic acid. SI Figure 3. Phylogenetic tree showing sequence accession numbers for the enzymes analyzed. SI Figure 4. SDS‐PAGE gels analyzing protein expression in Escherichia coli. SI Figure 5. Initial tests assessing thermostability of all enzymes from cell cultures. SI Figure 6. End‐point hydrolysis products for the polyesters tested in this study. SI Figure 7. Measurement of bulk UV absorbance to detect PBAT degradation. SI Figure 8. EqAD activity measured in 185 μL of supernatant from a reaction containing 100 nM Cl_EstA and 5 mg/mL PBAT, incubated at 40°C for 48 hours. SI Table 1. Gene sequences for the proteins expressed in this study. SI Table 2. Statistical comparison of residual activity at key temperatures (69.25°C, 78.00°C, and 84.25°C) shown in Figure 4. Newly identified enzymes were compared to Cl_EstA using Welch's two‐tailed t‐tests (unequal variance, n = 3). [file MBO3-14-e70144-s001.docx]

**Supplementary Information**


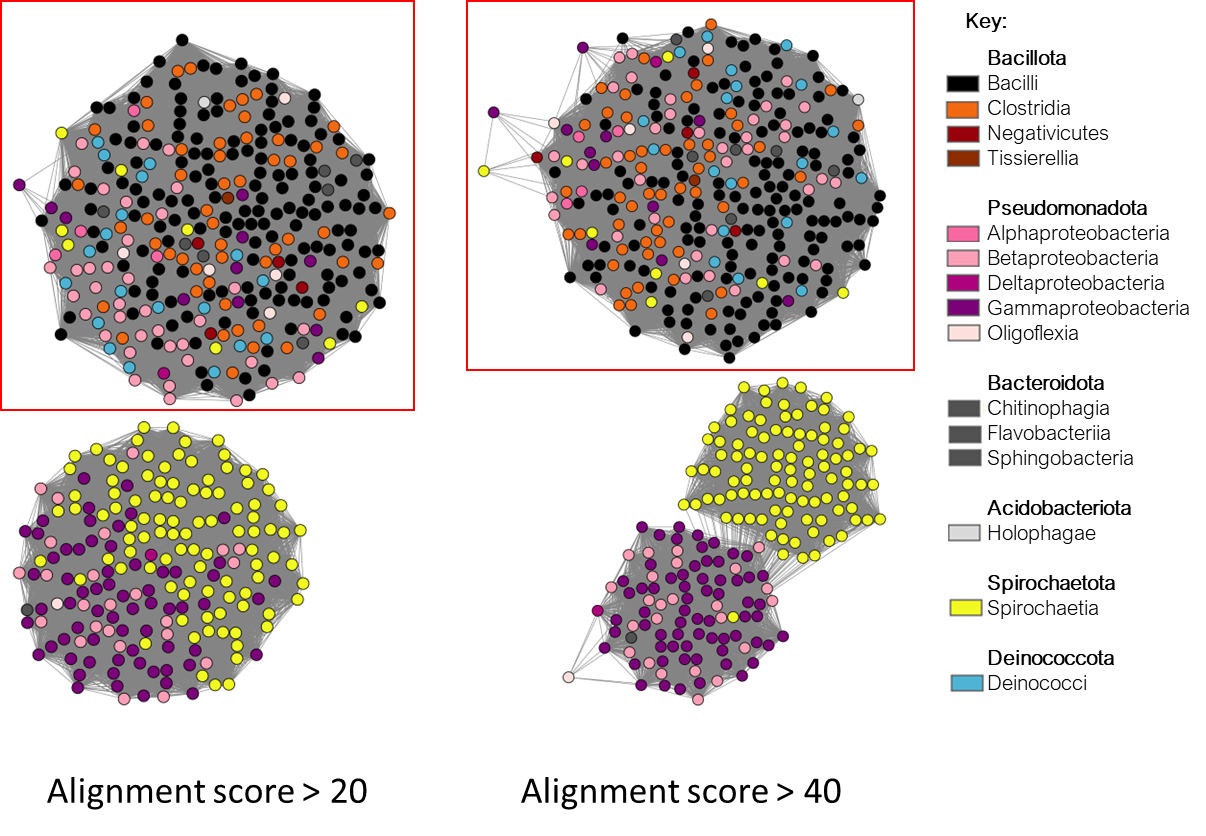


**SI Figure 1:** Initial prediction of iso-functional homologues of Cl_EstA, Cl_EstB and PfL1 using a sequence similarity network (SSN). The selected sequences clustered together even with a large jump in alignment score cut-off value from 20 to 40. Nodes represent individual proteins and are coloured based on the taxonomy class of the source organism. The alignment score is represented by the edges, such that shorter edges represent higher scores, causing clusters of proteins (nodes) that are similar to each other.

**
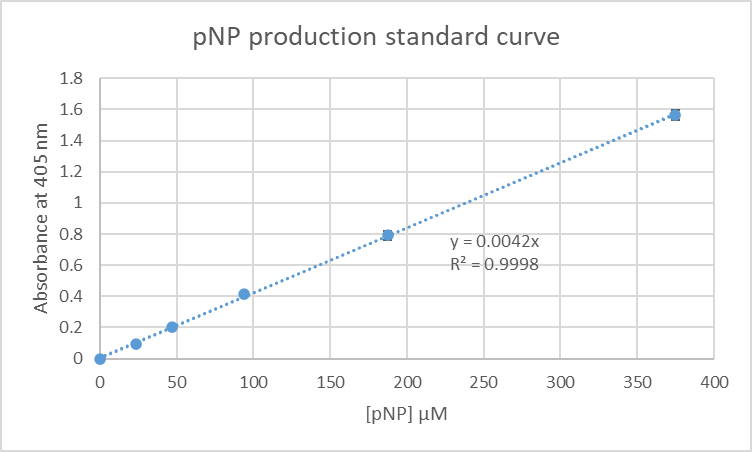
**

**
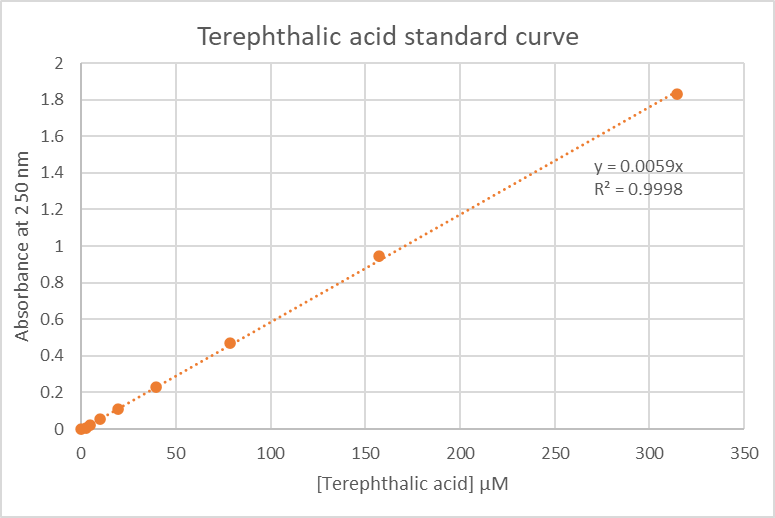
**

**SI Figure 2:** Standard curves for p-nitrophenol (pNP) and terephthalic acid used in this work. Reaction volume for pNP was 100 μL and terephthalic was 200 μL

**A**


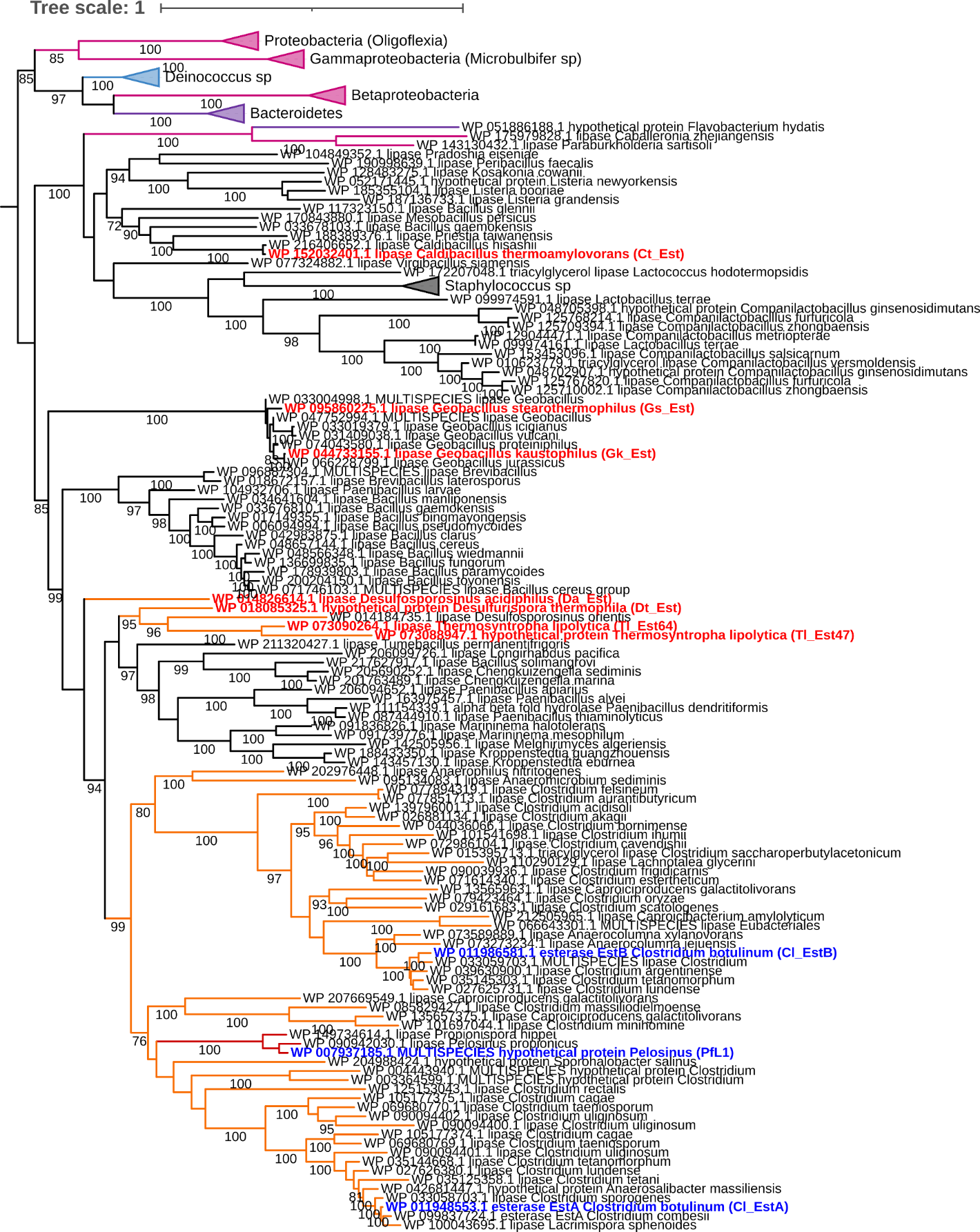


**B**
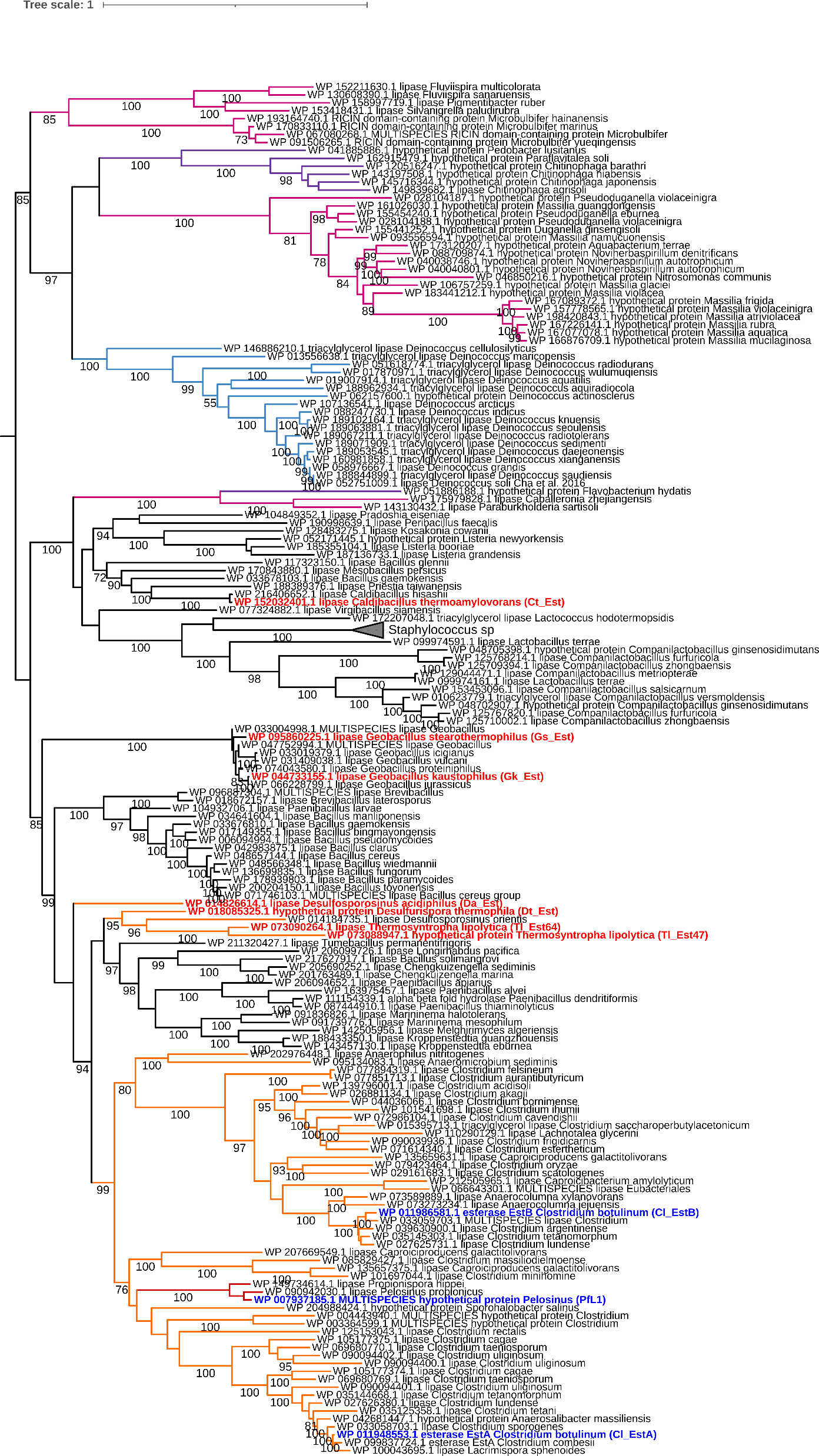


**SI figure 3.**  Phylogenetic tree containing sequence accession numbers. **A.** Similar tree view as in figure 2 with more detailed sequence labelling. **B.** Expanded view of the collapsed clades in panel A and in Figure 2. In both panels, UF-Boot values are shown for nodes with SH-aLRT support values >= 80%. Branches are coloured as follows: Clostridia - orange, Bacilli - black, Negetivicutes - red, Psuedomonadota – pink, Bacteroidota – purple, Deinococcota – light blue.


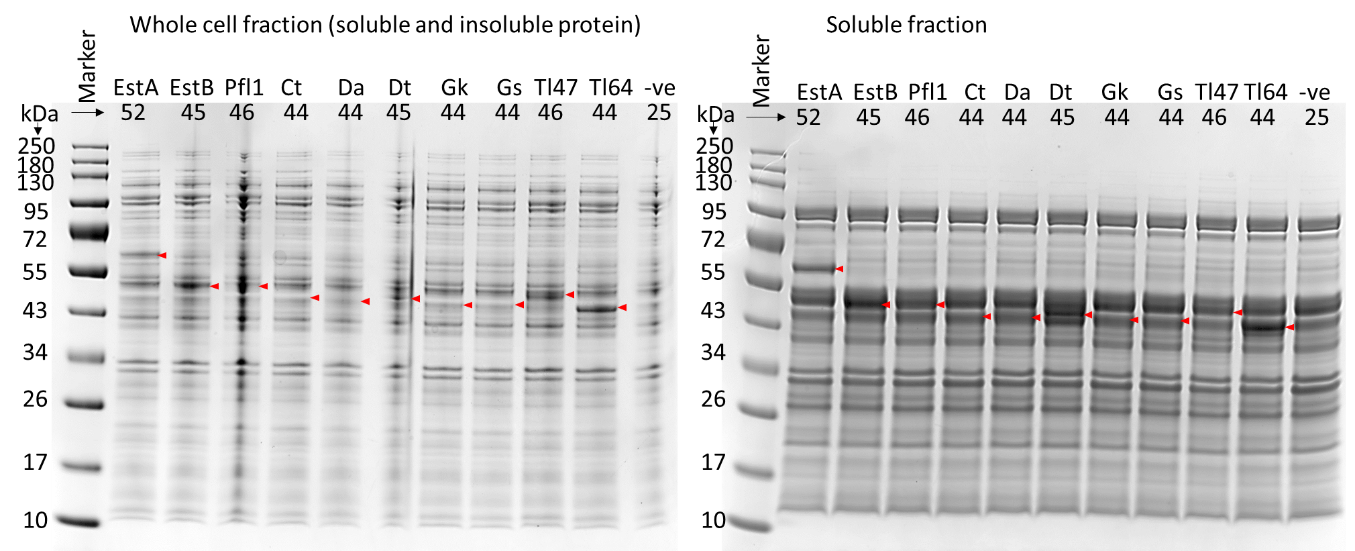


**SI Figure 4.** SDS-PAGE gels analysing protein expression in *Escherichia coli.* The gel on the left shows samples run from the whole cell preparations and the gel on the right shows samples from only the soluble protein fraction of the cells. A marker is shown as well as the expected sizes of the bands of each protein.


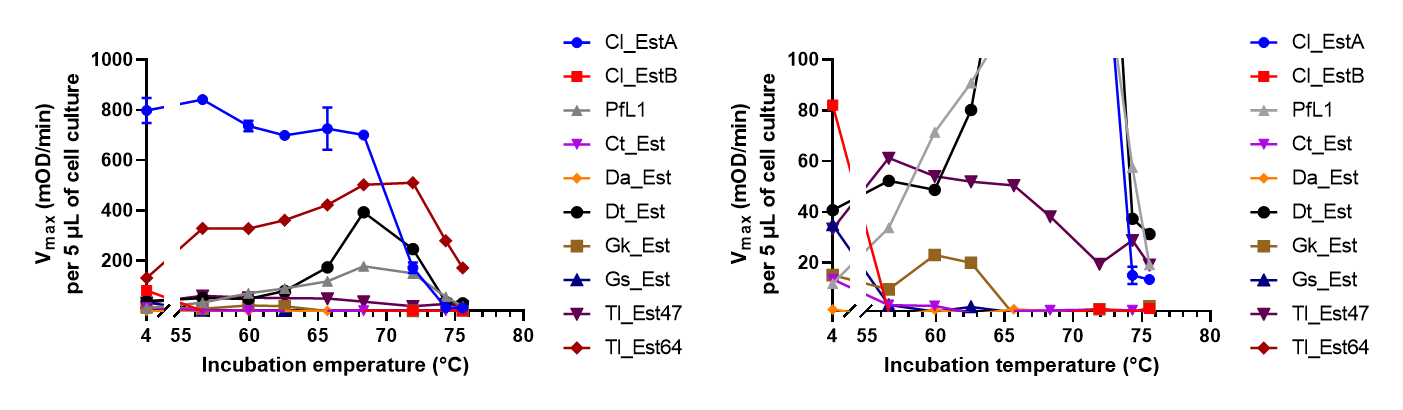


**SI Figure 5.** Initial tests checking the thermostability of all the enzymes from cell cultures.


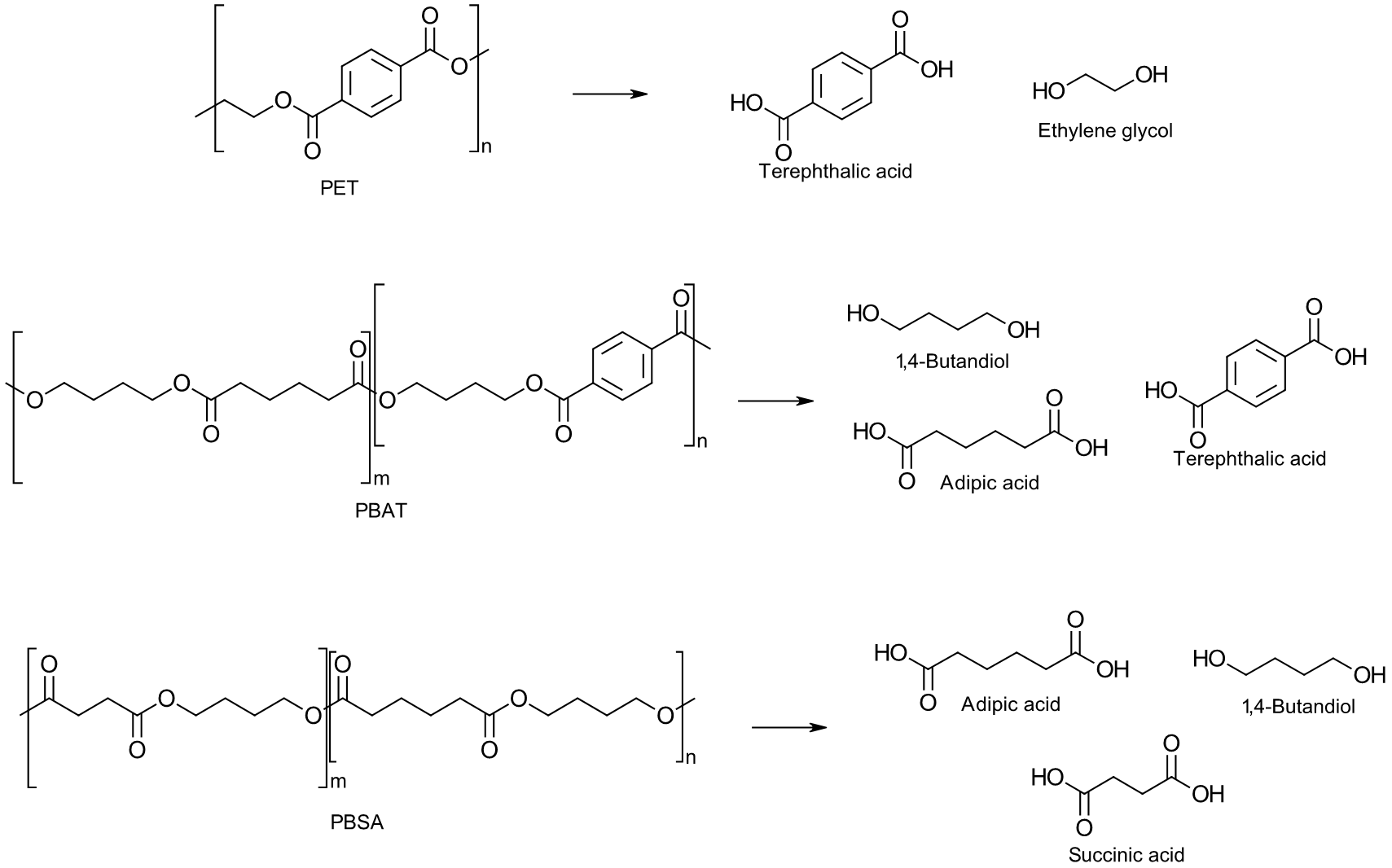


**SI Figure 6.** End-point hydrolysis products for the polyesters tested in this study.


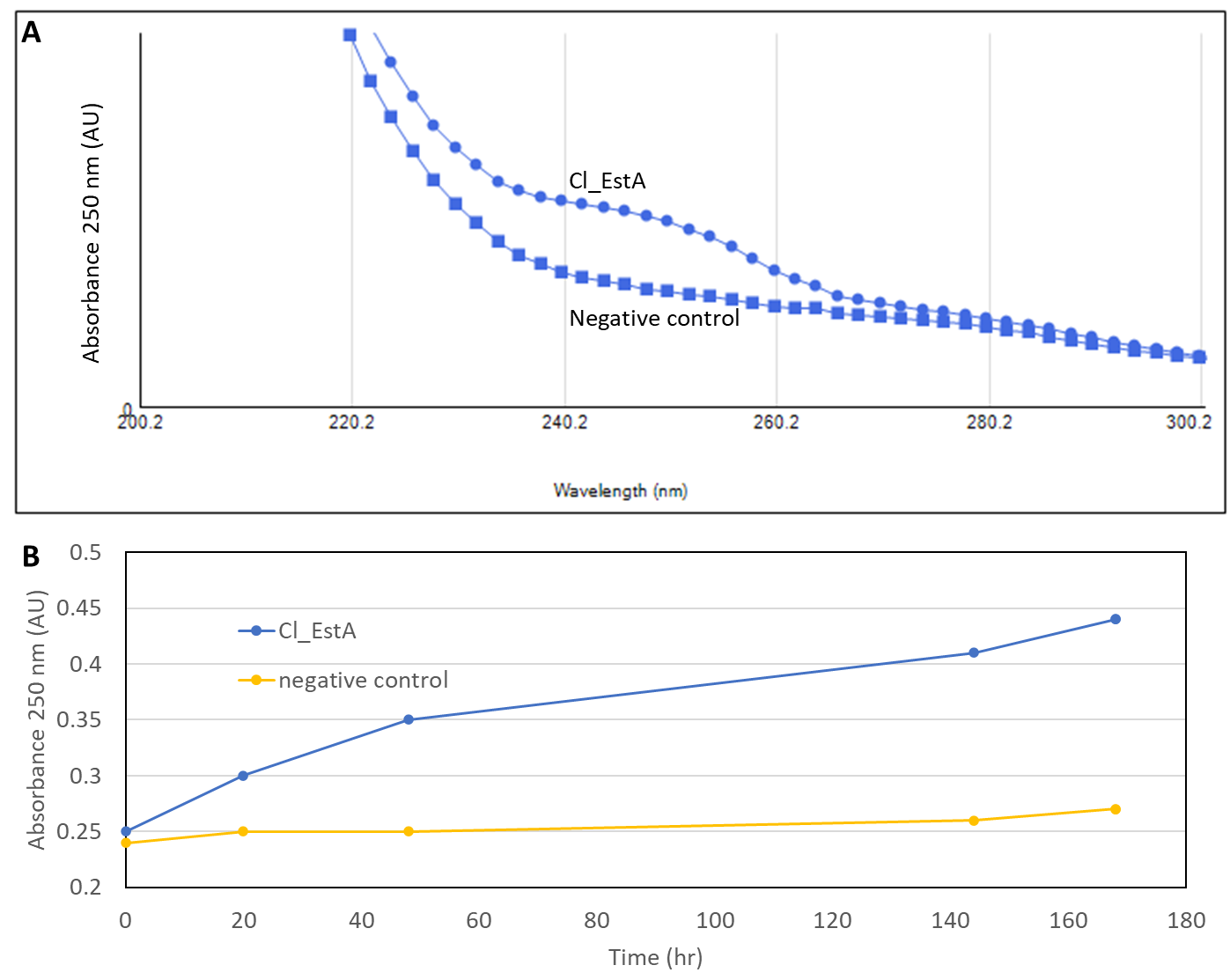


**SI Figure 7.** Measuring ‘bulk’ UV absorbance to detect PBAT degradation. Reactions contained 100 µL of cell culture expressing Cl_EstA or the negative control plasmid and ~5 mg/mL PBAT per 1 mL.


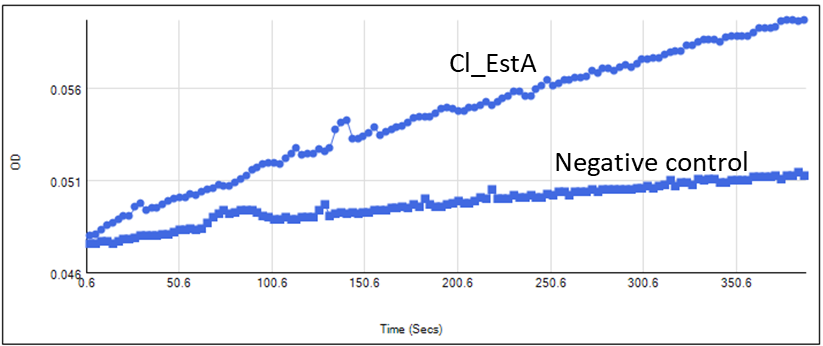


**SI Figure 8.** EqAD activity on 185 µl of supernatant from a reaction containing 100 nM Cl_EstA and 5 mg/mL PBAT that was incubated at 40 °C for 48 hr. The EqAD reaction contained 250 µM NAD^+^ and 0.1 U/mL of EqAD in a final reaction volume of 200 µL, and the reaction progress was measured by following the change in absorbance at 340 nm upon NADH^+^+H^+^ production.

**SI Table 1.** Gene sequences for the proteins expressed in this study. All genes were cloned into the *E. coli* expression vector pET-29b(+) between the Nde1 and Xho1 restriction enzyme sites, such as to introduce a C-terminal 6xHistidine tag

| plasmid name | Insert abbreviation | Insert sequence (including the Nde1 and Xho1 sites on vector) |
| --- | --- | --- |
| C.thermoamylovorans_pET-29b(+) | Ct_Est | CATATGGAAACCAGCGGTAACGACTACCCGATTGTGCTGGTTCACGGCCTGGGTGGCTGGGGCAAGGGTGAGTTCCTGGGTTATCGTTACTGGGGTGGCCTGAAGGATATTGAATTTTACCTGAACCAGACCGGTCACCGTACCTACGTGGCGACCGTTGGTCCGGTGAGCAGCAACTGGGACCGTGCGGTTGAGCTGTACTATTACATCAAGGGTGGCACCGTGGATTATGGTGCGGCGCATGCGAAAGAACACGGTCACGCGCGTTTCGGCCGTACCTATCCGGGTATTTACGGCCAGTGGGACGAGACCAACAAGATCCACCTGATTGGTCACAGCATGGGTGGCCAAACCAGCCGTATGCTGGTTGAACTGCTGAAGAGCGGTAGCCAGAAAGAGCAAGAATATTACAGCCAACACCCGGAGGAAGGCATCAGCCCGCTGTTCACCGGTGGCAAAAACTGGGTTCATAGCGTGACCAGCCTGGCGACCCCGCACAACGGTAGCACCTTTGCGGACCAGGAGCAAATCGTGAGCTTCATTAAGGATTTTATCATTCACCTGGCGAGCGCGGCGGGCCAGAAACAAGAAAGCCTGATCTATGACTTCAAGCTGGATCAGTGGGGTCTGAAACGTCAACCGGGCGAGAGCTTTCACGCGTACATGAACCGTGTTATGACCAGCCCGATCTGGCAAAGCAACGACATTAGCGCGTATGATCTGACCACCCTGGGTGCGCAGGAACTGAACCAATGGATGAAGACCTACCCGGATGTGTATTACCTGAGCTATACCGGCAACGCGAGCTACCGTGGTGTGGTTACCGGCAACTATTACCCGATCGGCACCATGCACCCGCTGTTCACCCTGATTAGCATGCAGATGGGCAGCTACACCCGTCAAAGCCCGGCGCCGGTTATTGACCGTAGCTGGCTGCCGAACGATGGTATTGTGAACGTGGTTAGCGCGAAGTATCCGTTTGGTCACCCGAACAGCCCGTACGACGGTGCGATCAAACAGGGCGTTTGGAACAGCTTTCCGGTGATGGAGGGTTGGGACCACATGGATTTCATCAACTTTATTGGTAGCAACACCCCGGGCTATTTTAGCATTTACGGCTATTACAACGATGTTGCGAACCGTGTGCACAGCCTGCCGAAACTCGAG |
| g.kaustophilus_pET-29b(+) | Gk_Est | CATATGGCGGCGAGCCGTGCGAACGATGCGCCGATTGTTCTGCTGCACGGCTTCACCGGTTGGGGTCGTGAGGAAATGTTCGGCTTTAAGTACTGGGGTGGCGTGCGTGGTGATATTGAGCAGCTGCTGAACGCGCAAGGCTACCGTACCTATACCCTGGCGGTTGGTCCGCTGAGCAGCAACTGGGACCGTGCGTGCGAAGCGTACGCGCAGCTGGTTGGTGGCACCGTTGATTATGGTGCGGCGCATGCGGCGAAGCATGGTCATGCGCGTTTTGGTCGTACCTACCCGGGTCTGCTGCCGGAGCTGAAACGTGGTGGCCGTGTGCACATCATTGCGCACAGCCAGGGTGGCCAAACCGCGCGTATGCTGGTTAGCCTGCTGGAGAACGGCAGCAAAGAGGAGCGTGAATATGCGAAAGCGCACAACGTGAGCCTGAGCCCGCTGTTCGAAGGTGGCCACAACTTTGTGCTGAGCGTTACCACCATTGCGACCCCGCACGATGGCACCACCCTGGTGAACATGGTTGACTTCACCGATCGTTTCTTTGATCTGCAGAAAGCGGTGCTGAAAGCTGCGGCGGTGGCGAGCAACGTTCCGTACACCAGCCAAGTTTATGACTTTAAGCTGGATCAGTGGGGCCTGCGTCGTCAACCGGGCGAGAGCTTCGACCAGTACTTTAAGCGTCTGAAACAAAGCCCGGTTTGGACCAGCGCGGACACCGCGCGTTATGATCTGAGCGTGCCGGGTGCGGAAGCGCTGAACCAGTGGGTTCAAGCGAGCCCGAACACCTACTATCTGAGCTTCGCGACCGAGCGTACCTACCGTGGTGCGCTGACCGGCAACTACTATCCGGAACTGGGCATGAACGTGTTCAGCGCGGCGGTTTGCGCGCCGTTTCTGGGCAGCTATCGTAACGCGGCGCTGGGTATCGACGATCGTTGGCTGGAGAACGATGGTATTGTGAACACCTTTAGCATGAACGGTCCGAAACGTGGCAGCACCGACCGTATTGTTCCGTACGATGGCACCCTGAAGAAAGGTGTGTGGAACGACATGGGCACCTATAACGTTGATCACCTGGAAGTGATCGGTGTTGACCCGAACCCGCTGTTCGATATTCACGCGTTTTACCTGCGTCTGGCGGAACAGCTGGCGAGCCTGCAACCGCTCGAG |
| g.stearothermophilus_pET-29b(+) | Gs_Est | CATATGGCGAGCCCGCGTGCGAACGATGCGCCGATCGTGCTGCTGCACGGTTTCACCGGTTGGGGCCGTGAGGAAATGCTGGGCTTTAAATACTGGGGTGGCGTGCGTGGTGACATTGAGCAGTGGCTGAACGATAACGGCTACCGTACCTATACCCTGGCGGTTGGTCCGCTGAGCAGCAACTGGGACCGTGCGTGCGAAGCGTACGCGCAACTGGTGGGTGGCACCGTTGATTATGGTGCGGCGCATGCGGCGAAGCATGGTCATGCGCGTTTCGGCCGTACCTACCTGGGTCTGCTGCCGGAGCTGAAACGTGGTGGCCGTGTGCACATCATTGCGCACAGCCAGGGTGGCCAAACCGCGCGTATGCTGGTTAGCCTGCTGGAGAACGGTAGCCAGGAAGAGCGTGAGTATGCGAAGGAACACAACGTGAGCCTGAGCCCGCTGTTCGAAGGTGGCCACCGTTTTGTGCTGAGCGTTACCACCATTGCGACCCCGCACGATGGCACCACCCTGGTGAACATGGTTGACTTCACCGATCGTTTCTTTGATCTGCAGAAAGCGGTGCTGGAGGCGGTGGCGGTTGCGAGCAACGTTCCGTACACCAGCCAAGGTTATGACTTTAAACTGGATCAGTGGGGCCTGCGTCGTCAACTGGGCGAGAGCTTCGATCACTACTTTGAACGTCTGAAGCGTAGCCCGGTTTGGACCAGCACCGACACCGCGCGTTATGATCTGAGCGTGAGCGGTGCGGAAAAGCTGAACCAATGGGTTAAAGCGAGCCCGAACACCTACTATCTGAGCTTTAGCACCGAGCGTACCTACCGTGGTGCGCTGACCGGCAACTACTATCCGGAACTGGGCATGAACGCGTTCAGCGCGATCGTGTGCGCGCCGTTTCTGGGCAGCTATCGTAACGCGGCGCTGGGTATTGACAGCCACTGGCTGGAAAACGATGGTATCGTTAACACCATTAGCATGAACGGTCCGAAACGTGGCAGCAACGACCGTATCGTGCCGTACGATGGCGCGCTGAAGAAAGGTGTTTGGAACGACATGGGCACCTATAACGTGGATCACCTGGAGATCATTGGTGTTGACCCGAACCCGAGCTTCGATATTCGTGCGTTTTACCTGCGTCTGGCGGAACAGCTGGTTAGCCTGCGTCCGCTCGAG |
| t.lipolytica64_pET-29b(+) | Tl_Est64 | CATATGCAGGAGATCAGCAGCAACAACAAGTATCCGATTGTGCTGGTTCACGGTCTGGGTGGCTTCGGCCGTGATGAAATGCTGGGCTTTAAGTACTGGGGTGGCCTGTATGACATCCAGGAGTACCTGAAAGCGCAAGGCTACGAAGTGTATACCGTGGCGGTTGGTCCGGTTAGCAGCAACTGGGATCGTGCGTGCGAGCTGTACGCGCAACTGGTGGGTGGCCGTGTTGACTATGGTGCGGCGCATGCGGCGAAGTATGGTCATGCGCGTTACGGCCGTACCTATGAGGGTCTGATCCCGGATCTGGGCAAAGTGGACCCGCAGACCGGTGAAGTGAAGAAAGTTCACCTGATTGGCCACAGCATGGGTGGCCAGACCGTGCGTACCCTGGTTCAACTGCTGGCGGAAGGTGATGCGGAGGAACGTAGCTTCCCGCAAGACAACATGAGCCCGCTGTTTGCGGGTGGCAACAACTGGGTGAAAAGCGTTACCACCATCAGCACCCCGCACGATGGCACCAGCCTGGACAACGCGATTAACAACGGCCTGCCGTGGCTGCAGAGCTTCGTTGGTTTTATGAGCACCCTGAGCACCCCGAACAGCCTGTATGATCTGAAGCTGGACCAGTGGGGCCTGACCCGTTGCCAAGGCGAGAAGGTGGCGGATTACCTGAAACGTGTTTTCAACAGCAGCTTTTGGAAAACCAGCCGTGATCTGAGCAACTGGGACCTGACCGTGGAGGGCGCGCGTGAACTGAACGGTTGGGTGAAGGCGCAACCGGACGTTTACTATTTCAGCTGGGCGACCAACGCGACCCGTAAAAGCCTGTTTAGCAACTACCAGGTTCCGATCCTGAGCATGAACCCGTTCCTGTATCCGTTTGCGCTGCACATCGGCAGCTACACCCGTAACATTCCGGGTCAGATCCCGATTGACAGCTCCTGGTGGCCGAACGATGGCCTGGTGAGCCTGATCAGCCAAAACGGTCCGAAGATTAACAGCACCGATACCATCATTAACTTCAACGGCAACCCGCAGCCGGGTGTGTGGAACTATATGGGTGTTATGGACACCTTTGATCACATGGACATCATTGGTATCGGCACCCTGTGGAACCCGTGCCCGTGGTATCTGGAGATTGCGAAACTGCTGACCAGCCTGCCGGCGCTCGAG |
| t.lipolytica47_pET-29b(+) | Tl_Est47 | CATATGAGCCAAAACTGCAACAGCAGCGGTAACAAGTACCCGATCGTGCTGGTTCACGGTCTGGGTGGCTTCGGCCGTGATGAACTGGGTGGCGTGATCAAGTATTGGGGTGGCGTTTACGACATTCAAGAATATCTGAAAAGCCGTGGTTATGAGGTGTACACCGTTAGCATTGGCCCGGTGAGCAGCAACTGGGATCGTGCGTGCGAGCTGTATGCGCAGCTGGTTGGTGGCACCGTTGACTATGGTGCGGCGCATGCGGCGAAGTATGGTCACAAACGTTATGGCCGTACCTACCCGGGTCTGATCCCGGATCTGGGCAAGATTGACCCGAAAACCGGTGAAATGAAGAAAGTGCACCTGATCGGTCACAGCATGGGTGGCCAGACCATTCGTACCCTGGTTCAACTGCTGGCGGAAGGCTGCGAGGAAGAGCGTAACTACAGCCAGGAGAACGTGAGCCCGCTGTTCATGGGTGGCAACAACTGGGTGAAGAGCGTTACCACCATCAGCACCCCGCACGATGGCACCAGCCTGGCGGATTACTATTACAACGACCCGAAAAGCGTTACCTGGCGTCAACACCTGATCGGCCTGATTGCGAGCACCCCGCTGAGCGACAACAGCAACCTGAAGTACGATCTGAAACTGGACCAGTGGGGTCTGACCTATAAGAAAGAAGAGAGCACCTATGAGTACCTGAAACGTATCATTAACAGCAGCTTTTGGATGACCAACCGTGATCTGGCGAACTACGACGTGAGCATCGAAGGCGCGGCGGAGCTGAACAGCTGGGTGAAGGCGCAGCCGGATGTTTATTACTTCAGCTGGGCGACCCAAGCGACCACCAAAAGCCGTTTTAGCGACTACCAGGTGCCGATTTGGAGCATGTATCTGGAAGGCTGGCAAAGCGCGTACTTCATGGGTAGCTATACCCGTAACATCCCGGGCAAGATTCCGGTTACCAGCGAGTGGTGGCCGAACGATGGTGCGGTGAACGTTATCAGCCAGAACGGCCCGAAAATTAACAGCAACGATCGTATCATTAACTTTGACGGCAACCCGGTGCCGGGTGTTTGGAACTTCATGGGTATCATGGACACCTTTGATCACCGTGACATCATTGGTCTGGGCACCTACTGGAACCCGTGCCCGTGGTATCTGAAGCACGTTGAACTGCTGGCGAAACTGCCGACCCTCGAG |
| d.thermophila_pET-29b(+) | Dt_Est | CATATGGCGGAGCGTCAGAACAACTACCCGATTGTGCTGGTTCACGGCTTCAGCGGTTGGGGCCGTGACGAAATGCTGGGTTTCAAGTATTGGGGTGGCTTTACCGATCTGCAGGAGAAACTGGAAAGCGCGGGCTACCAAACCTATACCGCGGCGGTGGGTCCGTTCAGCAGCAACTGGGACCGTGCGTGCGAGCTGTACGCGTTTATCAAGGGTGGCCGTGTTGATTACGGCAAGGCGCACGCGGCGAAATATGGTCACGCGCGTTACGGCCGTACCTATCCGGGTCTGCTGCCGAACTGGGGCGAAGTGGACCCGAGCACCGGCAAGACCGTTAAAATCCACCTGATTGGCCACAGCATGGGTGGCCAGACCATTCGTCTGCTGGCGCAACTGCTGGAGAACGGTGACCCGGATGAAATTGCGACCACCCCGCCGAGCGAGCTGAGCCCGCTGTTCAACGGCCAGAAGAAAAGCTGGATTCACAGCATTACCACCATCAGCACCCCGCACGATGGCACCACCCTGGCGGATGCGGTGAACGGTATGCTGCCGTTTGCGCAACAAACCGTGGCGCTGGTTGCGGCGGCGAGCGGTCTGTGCGCGGACAACCTGGTGTACGACTTCAAGCTGGATCAGTGGGGCCTGAAACGTCAACCGGGCGAGACCTTTGCGAGCTATGCGGACCGTGTTTGGAACAGCAGCATTTGGGAAAGCACCCACGACATCAGCGCGTGGGACCTGAGCCCGGATGGCGCGCGTGAGCTGAACAAGTGGGTTAAAGCGCAACCGGACATCTACTATTTCAGCTACGGCACCGAGGCGACCTTTCGTGAACTGATTACCGGTCACGAGATCCCGGAACTGAGCATGAACCCGATTTTCGTGCCGTTTGCGCTGCACATGGGCGCGTATACCCGTAACGTTCCGGACCGTGTGGTTATCGATAGCAACTGGTGGAAAAACGACGGTGTGGTTAACACCTGCAGCATGAGCGGTCCGCACATTGGCAGCAGCGATACCATCGTGAACTACAACGGCCAGGCGCAACCGGGTCGTTGGCACTATCTGGGTCTGCTGGAAAGCACCGACCACATGGATATTGTTGGTATCGGCACCCTGTGGAGCCCGACCAGCTGGTATCGTGACCTGGCGAGCCTGCTGGCGAGCCTGCCGGCGAACCTCGAG |
| d.acidiphilus_pET-29b(+) | Da_Est | CATATGATGAGCGAGCGTCAGAACGCGTATCCGATCGTTCTGGTGGTGGGTTTTGGTGGCTGGGATCGTAGCGAACTGCTGGGCTTCAAGTACTTTGGTGGCGTGAGCGACATTCAGGCGAACCTGACCAAAAGCGGTTATCAAACCTTCACCGCGGGTGTTGGCCCGTTTAGCAGCAACTGGGATCGTGCGTGCGAGCTGTACGCGATGCTGAAGGGTGGCGTGGTTGACTATGGTGCGGCGCATGCGGCGAAATATGGTCACGCGCGTTTCGGCGCGGATTTTGGTAGCGGCTACTATCCGCAGTGGAGCAACACCAACAAGGTGCACCTGGTTGGTCACAGCATGGGTGGCCAAACCAGCCGTCTGCTGACCCAGCTGCTGGAACAAGGCAGCAGCGAGGAACAGGCGTACGCGAACAGCCACCCGGGCACCGTTCTGAGCCCGCTGTTTGCGGGTGACAGCCACTGGGTGAAAAGCGTTACCACCATTGCGACCCCGAACAACGGCACCAGCCTGGCGATTGGCGTGACCAACCTGGTTCCGTACGCGCAGCAGCTGATTGCGTTTGCTGCGGCGGCGGCGGGTATTGCGAACGAGCCGCTGTATGACTTTAAGCTGGATCAATGGGGTGTGAAACGTGAGAGCGGCGAGAGCTTCAGCAGCTACAGCAACCGTGTTTGGAACAGCCAGATTTGGTGCAACACCCACGATATTAGCGCGTGGGACCTGTGCCCGGATGGTGCGAAGGAACTGAACAGCTGGGTGAAAGCGCAACCGGATGTTTACTATTTCAGCTGGGCGAACGACGCGACCTGGGAGGAACTGCTGACCGGCTATCAGCTGCCGGACGTGACCATGCTGCCGCTGTTCCAACCGTACGCGATCTTTATGGGTAGCTATACCCGTAACGACCCGGGCCACGTGGTTATTGATAGCCGTTGGTGGAAGAACGACGGTGTGGTTAACACCAACAGCATGGCGGGTCCGACCCTGGGCAGCAGCGACATCATTGTTAACTACAACGGCACCAGCCAGATCGGCAAGTGGAACTATATGGGCGAGAAAAGCGGCTGGGACCACGCGGATATGATCGGTATTGACACCAGCGATAGCCTGGGCTTCACCAACATCAACGACTTTTACAGCGAAATTGCGAACACCCTGGGTAGCCTGAAACTCGAG |
| Cl_EstA_pET-29b(+) | Cl_EstA | CATATGGCAGAACCCAAAGCCCAAGGAACCCAAAAGGTTGAATCGAGTACCACCAAAAAAGAAGTGAAAGATGCCGAAGAGACGATTAAGATCCCGACCTTAGAAGACATCGACAACCTGATCGATAGCGCGGAAGAAGTAAAAAGCGAGGAGGATATTAACAAGATGCCGCCGTTGAAGTTTCCAGTCGAATTTCCCGAAGTAAACACACGCTCGATCATTGGTGGCAACAATTACCCAATTGTGTTGGTGCATGGTTTTATGGGCTTTGGCCGCGATGAACTTCTGGGATACAAATACTGGGGCGGTGTGGTTGATCTGCAAGAGAAACTGAACGCCAGCGGTCATGAGACGTACACTGCCACAGTCGGCCCTGTGTCTAGCAACTGGGATCGGGCGTGTGAATTATACGCATATATCGTGGGCGGTACCGTTGACTACGGCGAGGCGCATGCGAAAAAATTCAAACACAATCGGTACGGTCGTACTTATCCAGGCATCTATAAAAACATTTCGAACGAGAACAAAATTCACCTGATCGGGCATTCGATGGGGGGTCAGACTATTCGCACGCTCACGCAGCTCCTGAGTGAAGGATCAGAAGAAGAGATTAACTGTGGCCAGGAAAATATCAGCCCGCTTTTTGAAGGCGGAAAACATTGGATCCACTCCGTCTCGACGATCTCTACGCCCAATGATGGAACGACCTTGAGCGATCTGATGCCGGCTAAAGATCTTATCAGCTATACGTTTGGGGTGCTCGGCACAATCACGGGGAAAAATAAGCTTTTTTCTTCAATTTATGACCTGAAACTGGACCAGTGGGGTTTGAAGAAACAAAATGGCGAATCACAGCGCGATTATATTGAACGTGTACTGGACAGTAACATCTGGAATTCAACGAAAGACATTGCGACCTATGACCTTAGTACCGAAGGTGCTCAGGAATTAAACACCTGGGTTAAAGCCCAACCGGATGTTTATTATTTCTCGTGGACCACCCAAGCAACGAAAGAATCTATCCTGACTGGTCACTCTGTGGCACAGATTGGTCCGATGAACCCTATCTTTTATCCTACCGCGAATCTGATGGGTCGTTACTCTCGCAACCAGAAAGATTTGCCGATCATTGATAAAAAATGGTTCCCCAATGATGGTGTGGTTAATTGCATTTCGCAGGATGGGCCCAAACTGGGGTCCAATGATGTGATCGAACAATATAACGGTGGTGTCAAAATTGGTCAATGGAATGCCATGCCCCGCATTATCAATACTGATCACATGGACATTGTGGGAACCTTCGGCAACGTCAAAGACTGGTATATGGACTATGCGTCTTTTCTCAGCAATTTGAGCCGTCTCGAG |
| Cl_EstB_pET-29b(+) | Cl_EstB | CATATGATGGCAGGTAATAGTGATGCTATGGGGGTGGGCAATAACTACCCTATCGTTATGGTGCACGGCTGCTTTGGTTGGGGGTCGAATGAGGGTGCGGGACTGTATTATTGGGGTGGCAAGGAATCTTTGACGCAAAAGCTGACGGAAAAAGGTTATACCGTGTACTCCCCTTCAATTGGCCCTGTTAGCAGCAACTGGGATCGTGCCTGCGAATTATACACTTATATTGTGGGCGGCACAGTAGATTATGGCGAATCCCACAGCAAAAAGTACGGCCATGAACGCTATGGGCGCTCATATCCGGGTGTTTATAAACAGATCGGCACGAAAGATTCGAGCGGTAACGTTCAGAAAATTCACTTGATTGGTCACAGCATGGGGGGACAGACGATTCGTCTGCTTGCGCAGCTGCTGGAAAATGGTGATCCTAACGAGTTATCATTTACCACCGATGGCTCCATTAACAGCCTCTTTACCGGAGGCAAATCGTGGGTGAGCTCAATCACCTCTATCGCTACACCTCATGATGGTTCCCAGGAAGCGCATATCAAGTGTGATATTGAACCGCTCACACATCAGTTCGTAGCGGCCATTGCGGCCATTAAAGGGAAAAACGTTAACCTGGATGATCTTAATTACGATTTCCAGTTGGACCAGTGGGGACTGAAACGTAAACCCGGGGAGTCGCGCCTCGCCTATAATAACCGTGTCATTAAAAGCGAAATTTGGAAAAAAACCAAAGATCTGAGTGTCTGGGATCTGAGTCCGGAGGGCGCGCGTGAATTTAACAGTTATGTGAAAGCGCAGAGCGATATTGATTACTTTAGCATTGCATGTGTTAACACGCATGAAGATAAACTGACCCATTTTCAGGTACCCAATAAAAAAATGAACCCAGTGCTGGTGAAATCTAGTATCTTTATGGGTATGTATACCAACAATAAATCCGGCGAGGTACCGATCGACAAATCTTGGTGGCGCAATGACGGCGTTGTGTCGGTCATCTCAGCGATCAACCCGAAAGTCGGTTCTACCGATAAAATTGTGGATTACTCTGGGACCGCAGTTAAAGGAACGTGGAATTATCTGGGCGAACTGGATAATACAGACCATATTGAAGTTTGTGGTATGAAGTACGACCGCAAAGGCATCGAACAAATGTATTTCAATGTGGCGGAAATGCTGAGCAAACTGCCGGTTGAACTCGAG |
| Pfl1_pET-29b(+) | Pfl1 | CATATGATGGACAGCGTGATGCAGACCAAAGAAATGGTTATGAAATCCAACGTTAACTCGTACCCGATCGTTCTCGTTCATGGCTTCATGGGCTGGGGCCGTAACGAGGTTCTGGGACTTAAATACTGGGGCGGCATCACCGATTACGAGCAAGAACTTAGCTCTTACGGCTATACCGCGTACACCGCCACGGTTGGTCCCGTTTCCTCAAACTGGGATCGTGCCTGTGAATTATACGCTTATATTAAAGGCGGTACTGTGGACTACGGACACGCTCACTCTACTCAGAAGGGTCATTCACGTTACGGCCGCACGTACCCAGGGCTCTACCCGGAATGGGGCAATCTGACTACCGAAGGCAAAGTTAACAAAATTCATTTAGTTGCCCACTCCATGGGCGGTCAGACCGTGCGTACCCTTGTCCAGCTGTTGAAAGAGGGCAGCGAGGAGGAACGCAACACCACCCCTTCTCAACTGTCCTCCCTCTTCGCAGGGGGTAAGAGCTGGGTACATAGCATTACCACCATCGCGAGTCCTCACGATGGTACCACCTTGGCAGATGGAATTAACATTTTTGGTGACTTTGCCAAAAACCTGGTGGCAAGCCTGGCATCCTTTACGGGTGCCGGGGAGAAACTGATCTACGATTTTAAACTGGATCAATGGGGTTTAAATCGTAAGAGCGGGGAATCTTTGACGGATTATACTAACCGTGTTTTTAACTCCGCGATTTGGAATAGCACCAACGACCTTGCAAATTGGGACCTGAGTACCGATGGTGCTCGTGTGTTGAACCAGTGGGTGAAAGCCCAATCCGATATTTACTATTTCTCTTATAGTACGTGTGCGACGGTTCCTTCTATTCTGACGTCCAACGAGCTGCCGCACGTGATTTACATGACGCCACTGCTGTATCCTTTTGGCCGTTTCATCGGGTCCTACACTCGTAACGAGCAGGGTCGCGTCATTATCGACAACTCCTGGAAACCCAACGATGGTGTGGTTAATACTATTAGTCAGAATGGTCCGAAAATTTGGAGCTCCGACAAAATCGTGAACTATAACGGGGTGCCGCAGATCGGTAAGTGGAATTCAATGCCGCTTTTAGATACAATTGATCACATGGACGCTTGTGGCATCGGTACGAACGCGCTGACACTGAGCTGGTATAAAGGTCTGGCGGAAAAACTGTCACAGCTGACCATTTCTAACCTCGAG |

**SI Table 2:** Statistical comparison of residual activity presented in (Figure 4) at key temperatures (69.25 °C, 78.00 °C, and 84.25 °C), where the newly identified enzymes are compared to Cl_EstA using Welch’s two-tailed *t*-tests (unequal variance, n = 3). Significance is indicated as: *p* < 0.05 (**), p < 0.01 (**), p < 0.001 (****), *ns* = not significant.

| **Temperature** | **Enzyme** | **Mean (%) ± Std** | ***t-*value vs EstA** | ***p*-value vs EstA** | **Significance** |
| --- | --- | --- | --- | --- | --- |
| **65.75 °C** | **EstA** | **88.69 ± 4.40** | **-** | **-** | **-** |
|  | dt | 603.30 ± 67.14 | -10.82 | 0.0082 | ** |
|  | gk | 23.28 ± 7.33 | 10.82 | 0.0011 | ** |
|  | Pfl1 | 625.22 ± 50.79 | -14.88 | 0.0042 | ** |
|  | tl47 | 74.52 ± 5.25 | 2.92 | 0.0447 | * |
|  | tl64 | 249.12 ± 10.43 | -20.05 | 0.0005 | *** |
| **69.25 °C** | **EstA** | **62.56 ± 10.59** | **-** | **-** | **-** |
|  | dt | 820.16 ± 98.89 | -10.77 | 0.0079 | ** |
|  | gk | 8.96 ± 3.94 | 6.71 | 0.0112 | * |
|  | Pfl1 | 749.51 ± 154.65 | -6.27 | 0.024 | * |
|  | tl47 | 55.45 ± 5.35 | 0.85 | 0.4599 | ns |
|  | tl64 | 327.21 ± 14.01 | -21.31 | 0 | *** |
| **78 °C** | **EstA** | **0.39 ± 0.10** | **-** | **-** | **-** |
|  | dt | 18.91 ± 2.18 | -11.98 | 0.0068 | ** |
|  | gk | 7.93 ± 3.88 | -2.75 | 0.1108 | ns |
|  | Pfl1 | 17.66 ± 1.25 | -19.53 | 0.0024 | ** |
|  | tl47 | 31.92 ± 5.46 | -8.16 | 0.0147 | * |
|  | tl64 | 18.45 ± 1.05 | -24.28 | 0.0015 | ** |
| **84.25 °C** | **EstA** | **0.29 ± 0.13** | **-** | **-** | **-** |
|  | dt | 5.12 ± 1.09 | -6.24 | 0.0231 | * |
|  | Pfl1 | 5.77 ± 3.11 | -2.49 | 0.1297 | ns |
|  | tl47 | 15.18 ± 1.14 | -18.29 | 0.0027 | ** |
|  | tl64 | 1.92 ± 0.81 | -2.82 | 0.1001 | ns |
